# Supplementary material for: Viral Infection Leads to a Unique Suite of Allelopathic Chemical Signals in Three Diatom Host–Virus Pairs
Source: Mar Drugs. 2024 May 17;22(5):228. doi: 10.3390/md22050228 (PMC11123003; doi:10.3390/md22050228)
Supplement: Supplementary file 1 [file marinedrugs-22-00228-s001.zip › Marine Drugs Supplemental Figures May 2024.pdf]

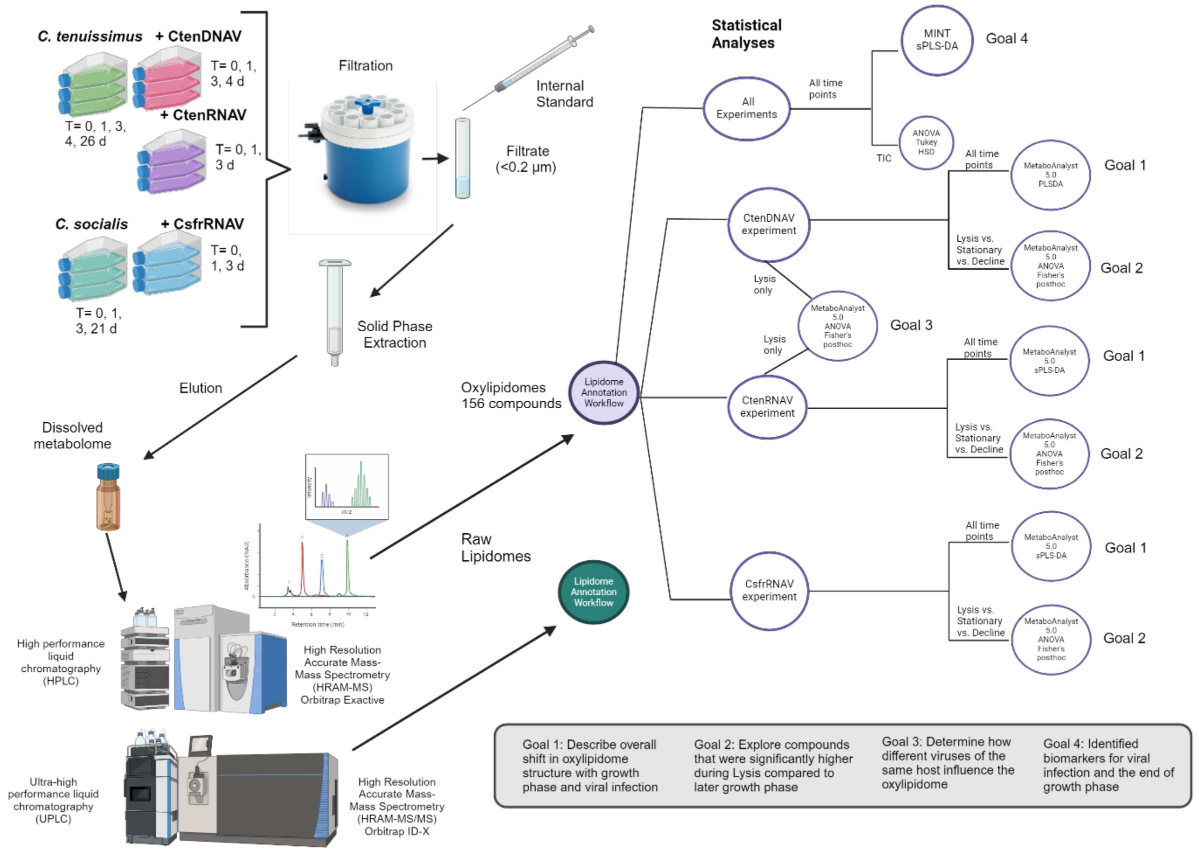

Figure S1. Experimental Design and workflow for the Chaetoceros-virus-oxylipin experiment. Chaetoceros spp. was grown in triplicate under two treatments, infected and uninfected, and were sampled at discrete timepoints for lipidomic analysis. A sample volume of 30 mL was filtered through a 0.2 um durapore, collecting the filtrate. The filtrate was spiked with a deuterated oxylipin internal standards (15-HETE-d8 to a final conc. of 10 µM) before being loaded onto a solid phase extraction cartridge. The dissolved metabolome was eluted off of the SPE cartridge and the sample was analyzed with HPLC-HRAM-MS in 2016 for quantitative analysis and UPLC-HRAM-MS^n in 2021 for ms2 verification of annotations. The raw lipidomes generated were annotated through our chemoinformatic pipelines, outlined in detail in Figure S10. The compounds annotated as free fatty acids and oxylipins within our dataset were defined as the oxylipidome. The 156 compounds in the oxylipidome were subject to a variety of statistical analyses on different subsets of the data as outlined above. Each of these analyses were aimed at the four goals outlined at the bottom of the diagram.

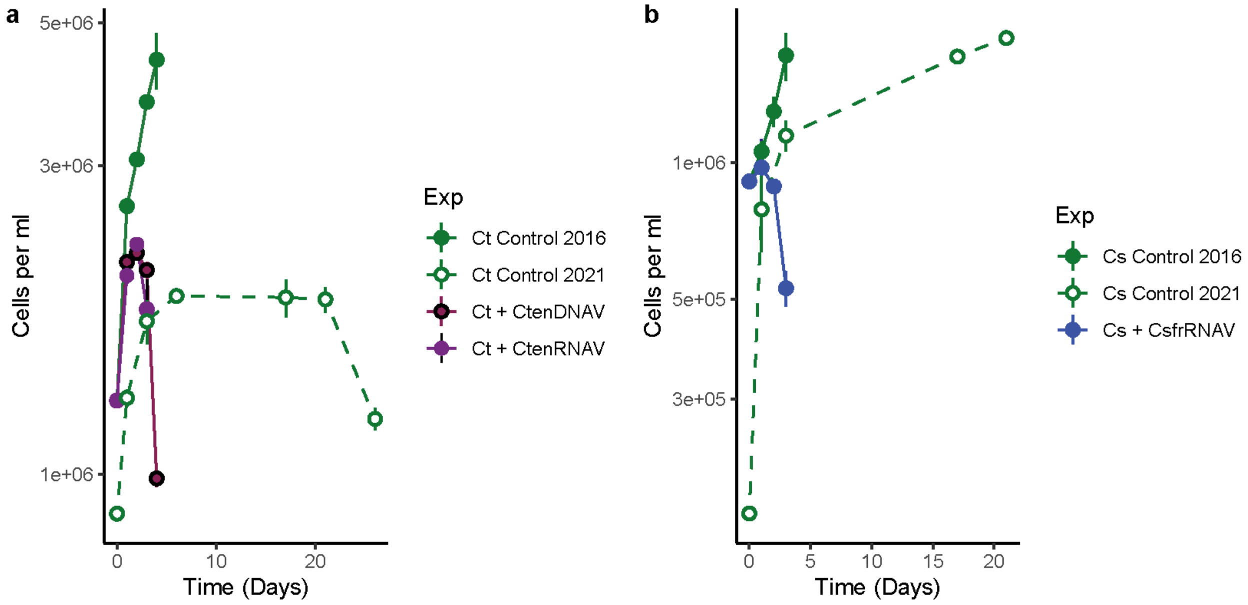

Figure S2. Time course of uninfected control and infected treatments for three diatom host-virus experiments. **a)** *C. tenuissimus* (Ct) concentration in the host-only control in 2016 (solid green, N=3) and 2021 (hollow green, N=3), and in cultures infected with CtenDNAV(magenta with black outline; N=3) and CtenRNAV (solid purple; N=3). **b)** C. socialis concentration in the host-only control in 2016 (solid green, N=3) and 2021 (hollow green, N=3), or in cultures infected with CsfrRNAV (blue; N=3). Discrepancies between the number of cells in the controls in 2016 vs. 2021 are likely a result of the different enumeration methods employed, microscopy in 2016 and flow cytometry in 2021. Error bars represent standard deviations.

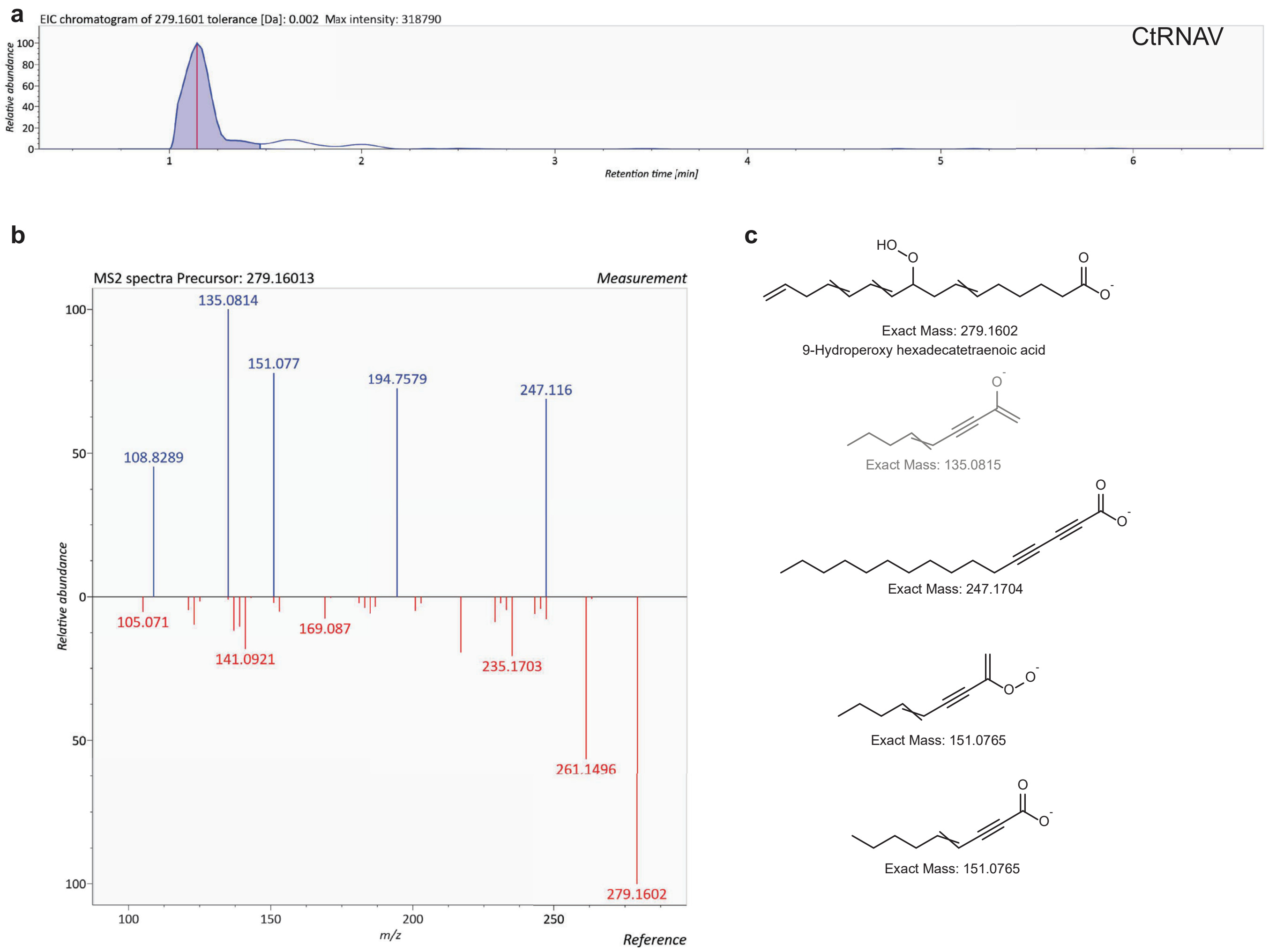

Figure S3. EIC and ms2 fragmentation for 9-HpHTE. **a)** Extracted ion chromatogram of the feature annotated as C16:4 oxylipin with 2 oxygens using exact mass and annotated to the positional level using ms2 fragmentation as 9-hydroperoxy hexadecatetraenoic acid (9-HpHTE) from the CtRNAv experiment. **b)** Comparison of the observed fragmentation for m/z= 279.16013 at RT = 1.14 minutes to the fragmentation of the strongest match in a boutique database of modeled oxylipin fragmentation (Supplemental File 1A). **c)** Chemical structure of the proposed parent Ion and fragments for the ms2 that match the putative annotation's modeled fragmentation.

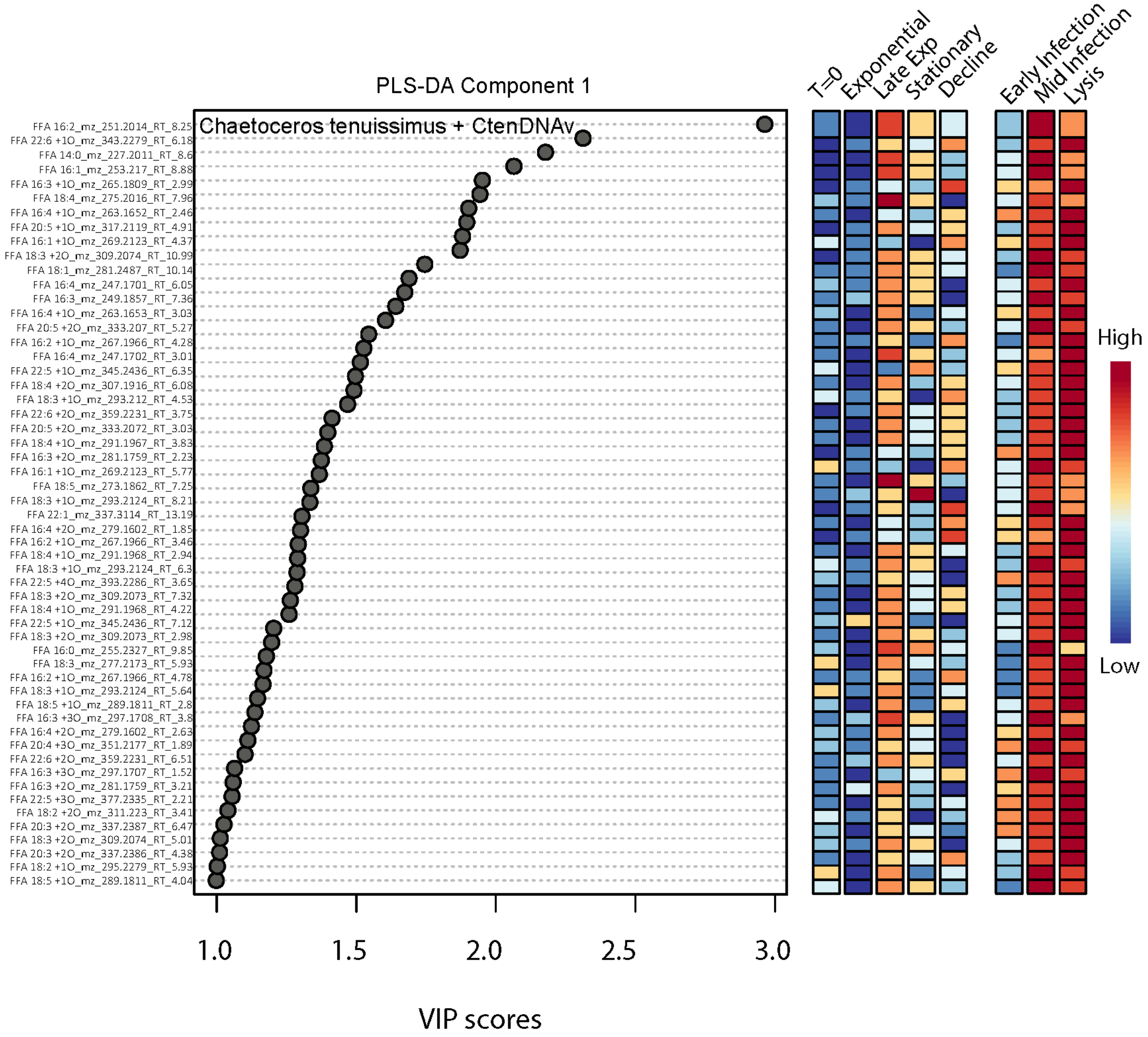

Figure S4. Relative abundance of compounds with variable importance scores higher than 1 for Component 1 of the PLS-DA of the CtenDNAV experiment presented in the main text in Figure 2a. Heatmap shows the relative abundance of each compound (log transformed and mean-centered) with red indicating an increase in concentration across the triplicates at that timepoint and blue indicating a decrease relative to the mean concentration of that compound across all samples.

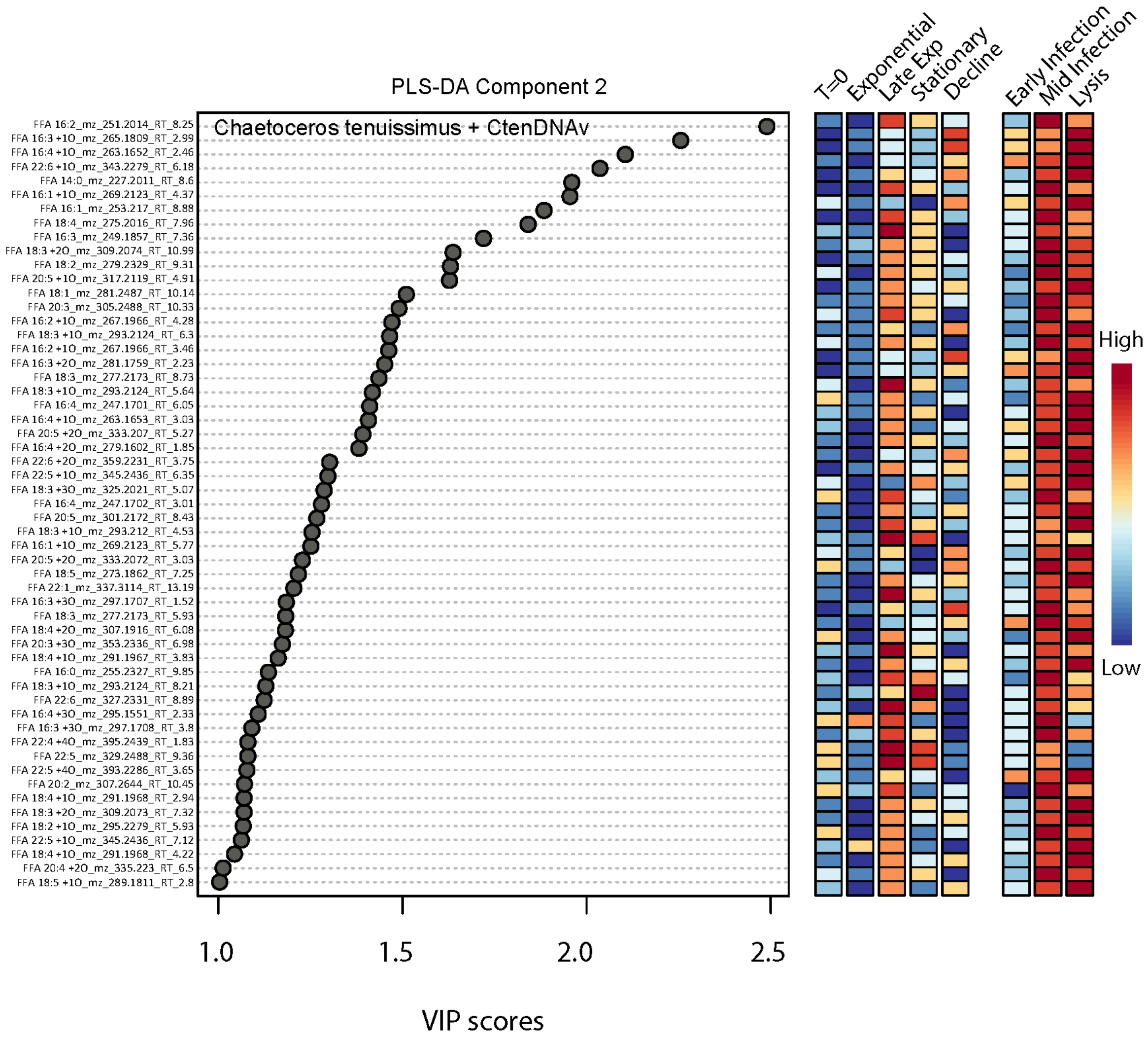

Figure S5. Relative abundance of compounds with variable importance scores higher than 1 for Component 2 of the PLS-DA of the CtenDNAV experiment presented in the main text in Figure 2a. Heatmap shows the relative abundance of each compound (log transformed and mean-centered) with red indicating an increase in concentration across the triplicates at that timepoint and blue indicating a decrease relative to the mean concentration of that compound across all samples.

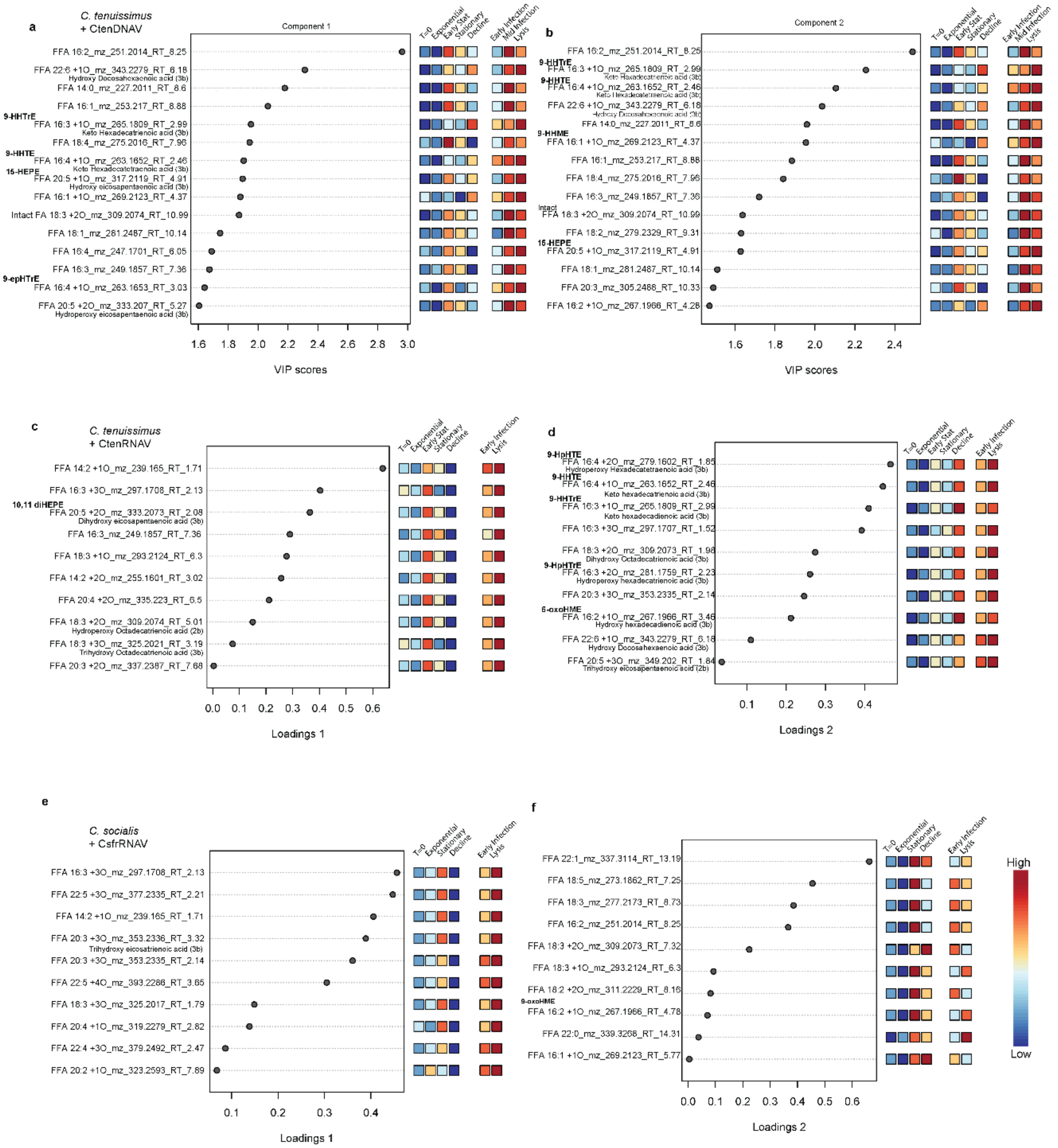

Figure S6. Heatmaps showing the average relative abundance of the significant compounds distinguishing between the virus infected treatments and the aging control treatments. Across all three diatom host-virus pairs, samples taken during virus lysis generally had the highest concentrations of the specific oxylipins, but most of the compounds were produced to some degree during the later phases of growth. Many compound were further annotated to the positional and functional-groups level, with *Chaetoceros tenuissimus* favoring 9-LOX pathways. **a-b)** The 15 most significant compounds structuring the *C. tenuissimus* + CtenDNAV experiment PLS-DA based on the variable importance to projections (VIP) scores (values > 1 considered significant; see Figure S3-S4 for all significant compounds) **c-d)** The 10 compounds used to derive Component 1 and 2 in the sPLS-DA for the CtenRNAV experiment. **e-f).** The 10 compounds were used to derive Component 1 and 2 in the sPLS-DA for the *C. socialis* + CsfrRNAV experiment.

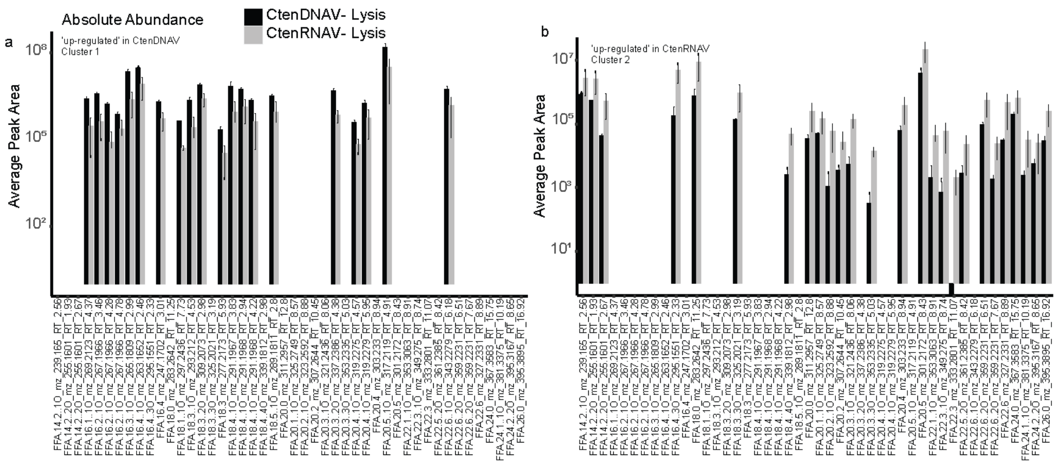

Figure S7. Difference in chain length of oxylipins that were significantly more abundant during lysis by ssDNA virus vs ssRNA virus in the *Chaetoceros tenuissimus*. **a)** Average peak area of each compound in Cluster 1 which were significantly more abundant in the CtenDNAV (black) experiments compared to the CtenRNAV (grey). Compounds are organized by chain length and double bond equivalent. (**b**) Average peak area of the compounds in Cluster 2, which were significantly more abundant in the CtenRNAV
(grey) experiments compared to the CtenDNAV (black). Compounds are organized by chain length and double bond equivalent.

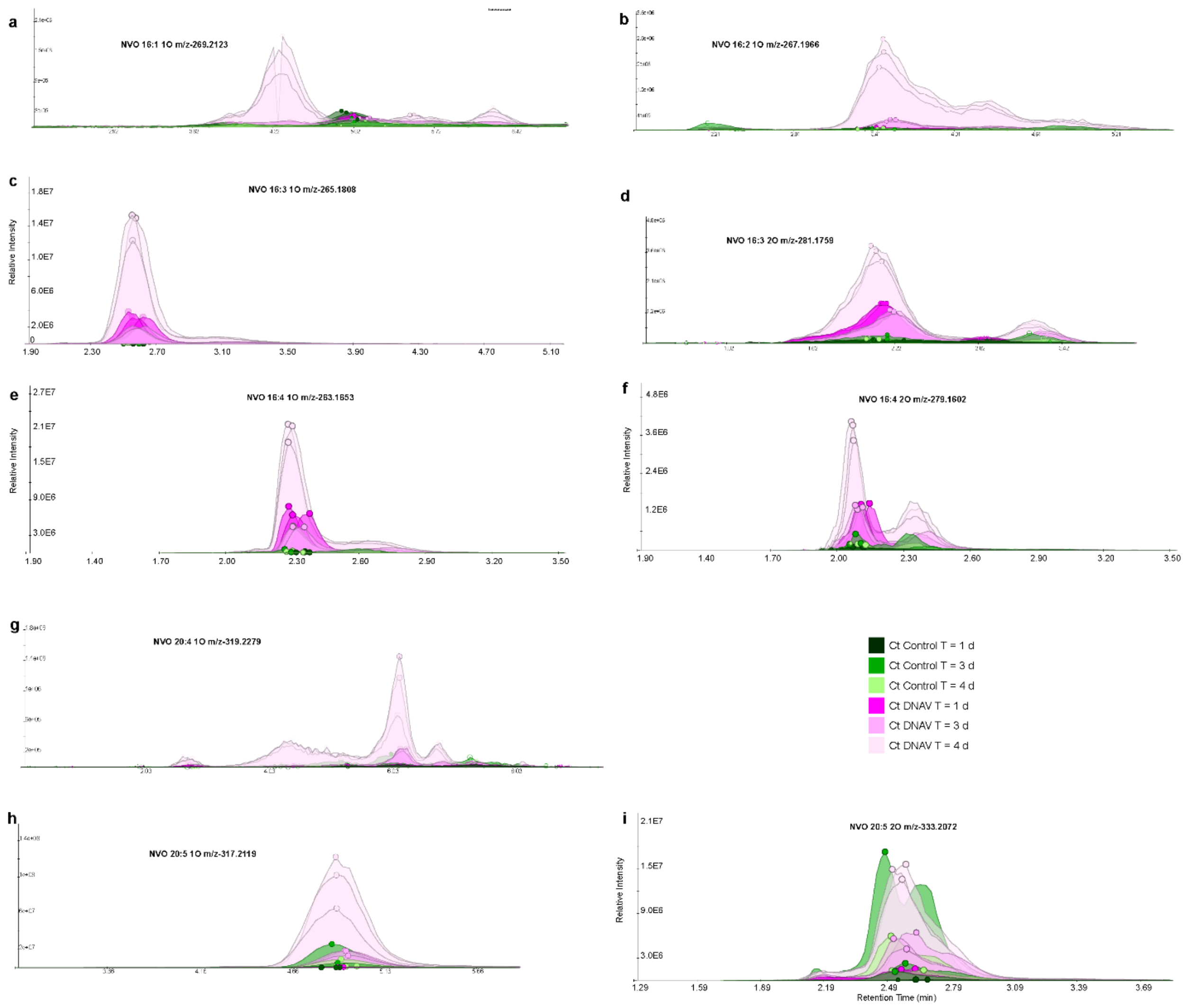

Figure S8. Extracted ion chromatogram for the homologs of allelopathic oxylipins identified as being significantly upregulated in dissolved lipidomes of *Chaetoceros tenuissimus* infected with CtenDNAV. Control treatments are presented in shades of green and infected treatments are presented in shades of pink, lighter shades correlate to later timepoints. Note that there are multiple isomers for each exact mass, as in multiple peaks at different retention times. The retention times alignment is slightly different in the program used to generate these plots (MAVEN) compared to the program integrated into our lipidomic pipeline (XCMS).

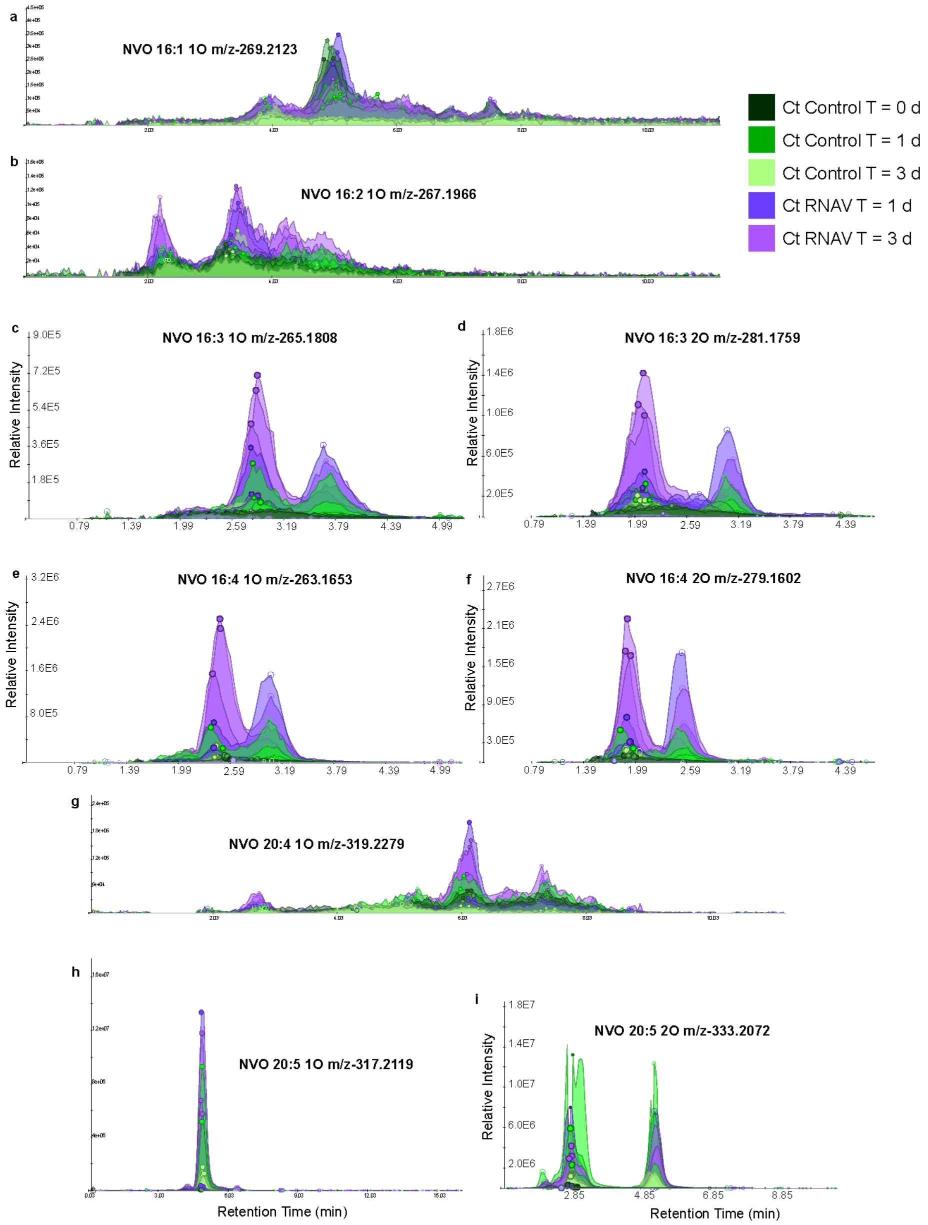

Figure S9. Extracted ion chromatogram for the homologs of allelopathic oxylipins identified as being significantly upregulated in dissolved lipidomes of *Chaetoceros tenuissimus* infected with CtenRNAV. Control treatments are presented in shades of green and infected treatments are presented in shades of purple, lighter shades correlate to later timepoints. Note that there are multiple isomers for each exact mass, as in multiple peaks at different retention times. The retention times alignment is slightly different in the program used to generate these plots (MAVEN) compared to the program integrated into our lipidomic pipeline (XCMS).

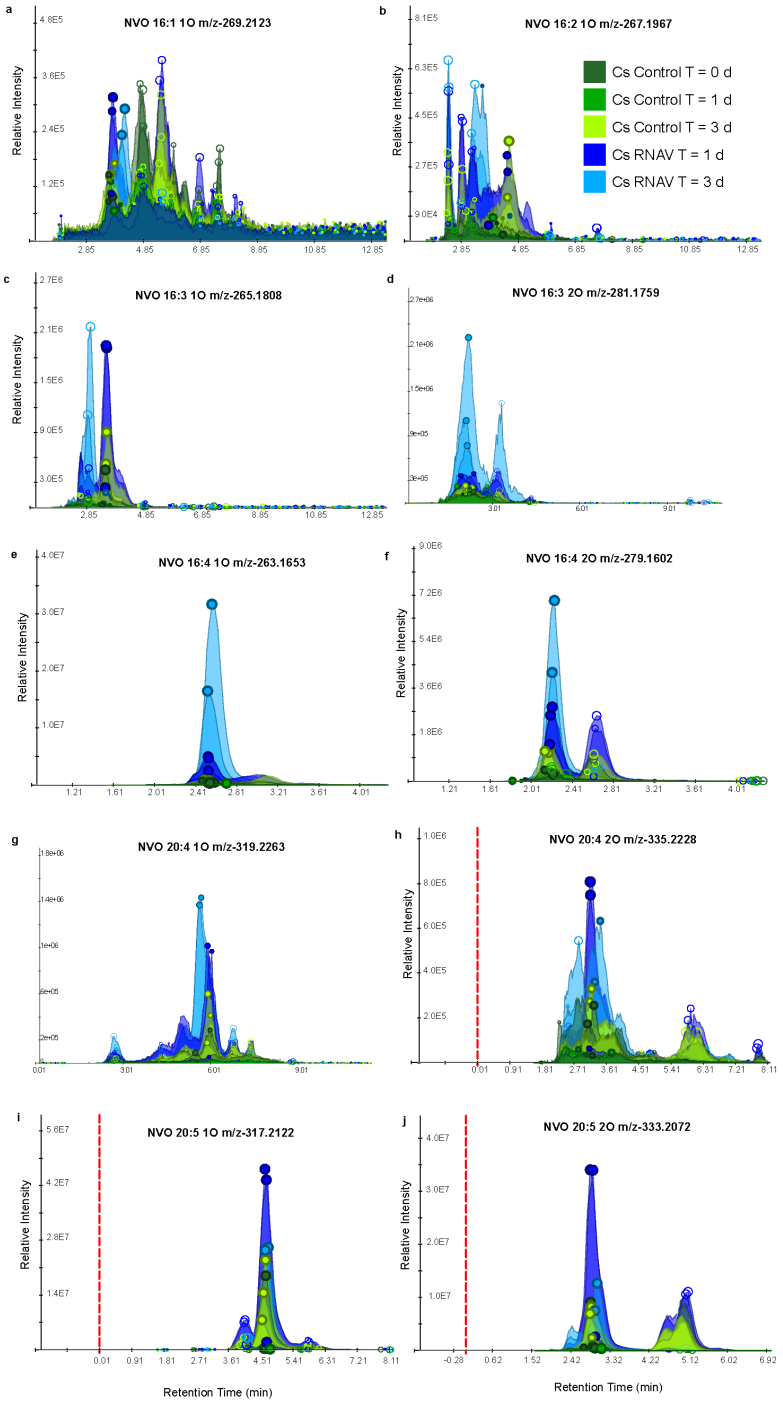

Figure S10. Extracted ion chromatogram for the homologs of allelopathic oxylipins identified as being significantly upregulated in dissolved lipidomes of *Chaetoceros socialis* infected with CsfrRNAV. Control treatments are presented in shades of green and infected treatments are presented in shades of blue, lighter shades correlate to later timepoints. Note that there are multiple peaks at different retention times for each exact mass, representing the production of several structural isomers. The retention times alignment is slightly different in the program used to generate these plots (MAVEN) compared to the program integrated into our lipidomic pipeline (XCMS).

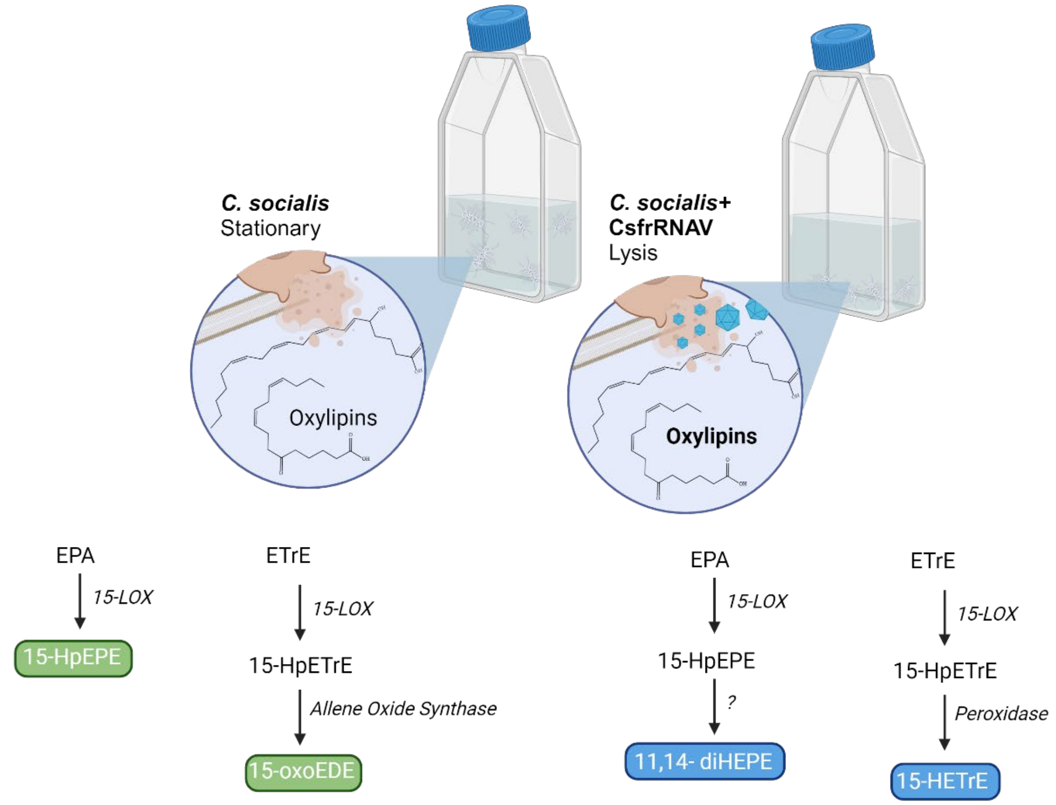

Figure S11. Divergence of 15-LOX oxylipin biosynthetic pathways observed in *Chaetoceros socialis* during stationary phase vs. during lysis from infection with CsfrRNAV. All the enzymes represent hypothesized biosynthetic pathways based on homology to plant systems (Andreou et al., 2009 and citations therein). During stationary phase growth, eicosapentaenoic acid (EPA) is oxygenated by 15-LOX to produce 15-hydroperoxy eicosapentaenoic acid (15-HpEPE), and eicosatrienoic acid is oxygenated by 15-LOX to produce 15-hydroperoxy eicosatrienoic acid (15-HpETrE) which is subsequently modified to 15-oxo eicosadienoic acid by an enzyme hypothesized to be allene oxide synthase. During viral lysis, 15-HpEPE is modified by an unknown cascade of enzymes and intermediate oxylipins to a 11,14-dihydroxy eicosapentaenoic acid, potentially through a hydroxyepoxy acid intermediate. 15-HpETrE is modified by a peroxidase to 15-hydroxy acid (15-HETrE).

a
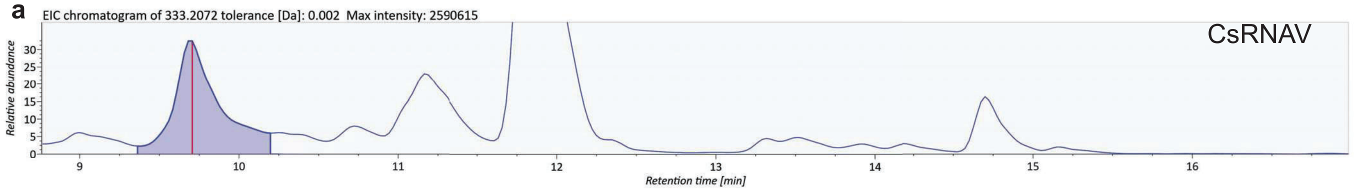

b
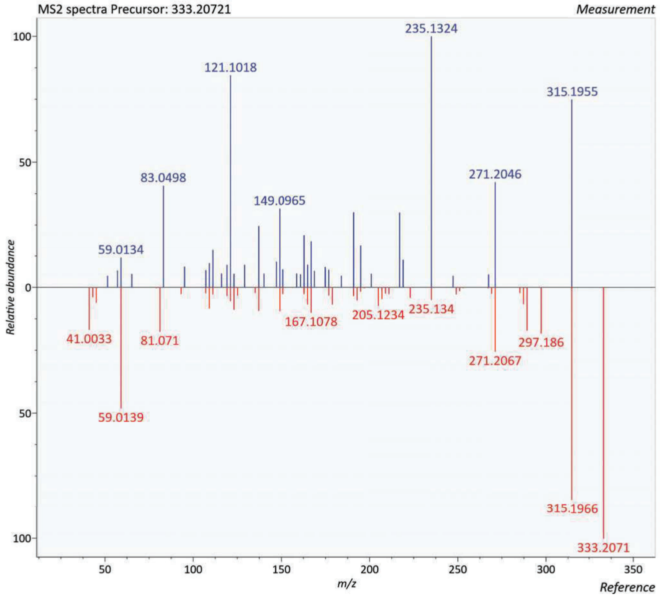

c
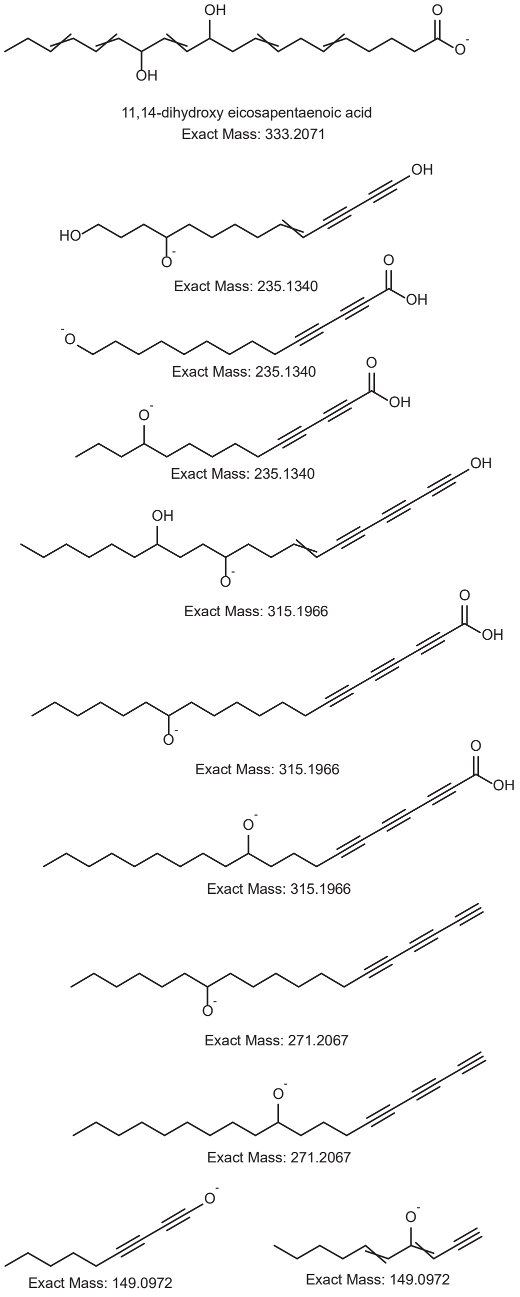

Figure S12. EIC and ms2 fragmentation for 11,14-diHEPE. **a)** Extracted ion chromatogram of the feature annotated as C20:5 oxylipin with 2 oxygens using exact mass and annotated to the positional level using ms2 fragmentation as 11,14- eicosapentaenoic acid (11,14-diHEPE) from the CsRNAv experiment. **b)** Comparison of the observed fragmentation for m/z= 333.20721 at RT = 2.08 minutes to the fragmentation of the strongest match in a boutique database of modeled oxylipin fragmentation (Supplemental File 1A). **c)** Chemical structure of the proposed parent Ion and fragments for the ms2 that match the putative annotation's modeled fragmentation.

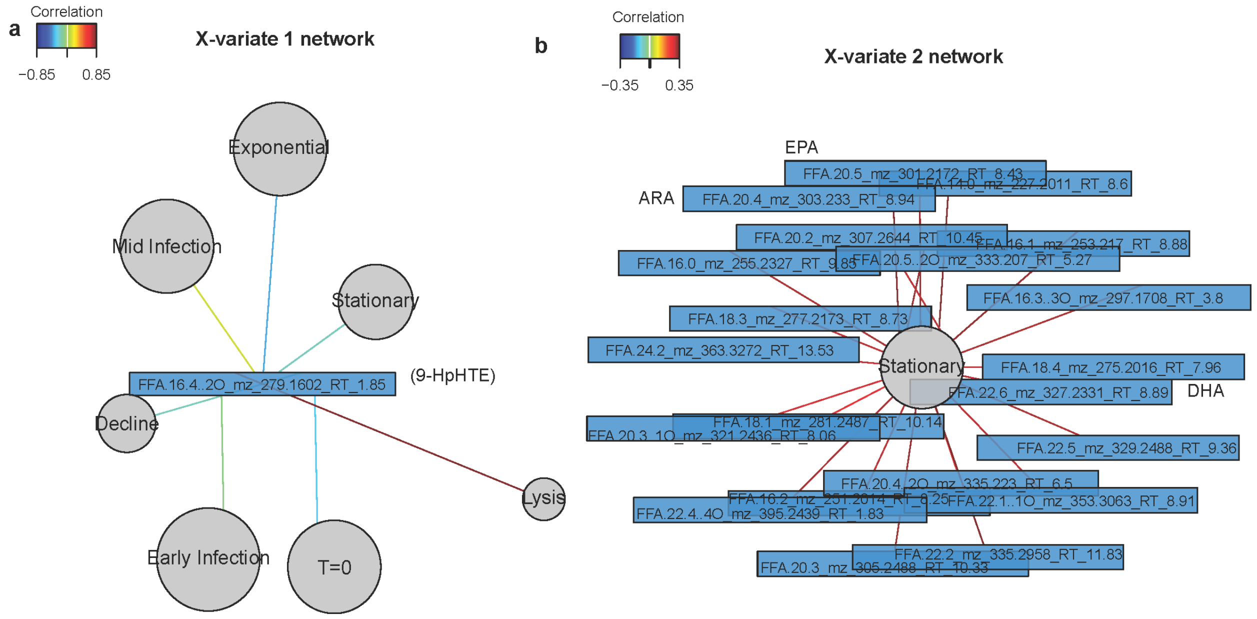

Figure S13. Correlation networks for peak area data. **(a)** Network analysis showing the correlations between the virally infected treatments and the compound correlated with X-variate 1 in the MINT sPLSDA of the Chaetoceros diatom host-virus lipidomes peak area data. **(b)** Network analysis showing the correlations between the Stationary control treatment and the compounds correlated with X-variate 2 in the sPLSDA.

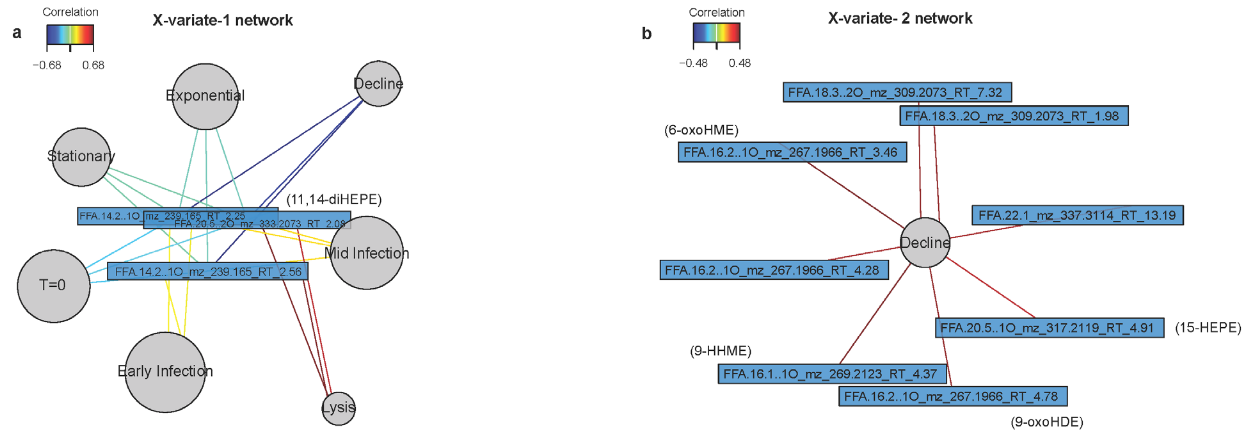

Figure S14. Correlation networks for relative abundance data. (**a**) Network analysis showing the correlations between the virally infected treatments and the compound correlated with X-variate 1 in the MINT sPLSDA of the Chaetoceros diatom host-virus lipidomes relative abundance data. (**b**) Network analysis showing the correlations between the Decline control treatment and the compounds correlated with X-variate 2 in the sPLSDA.

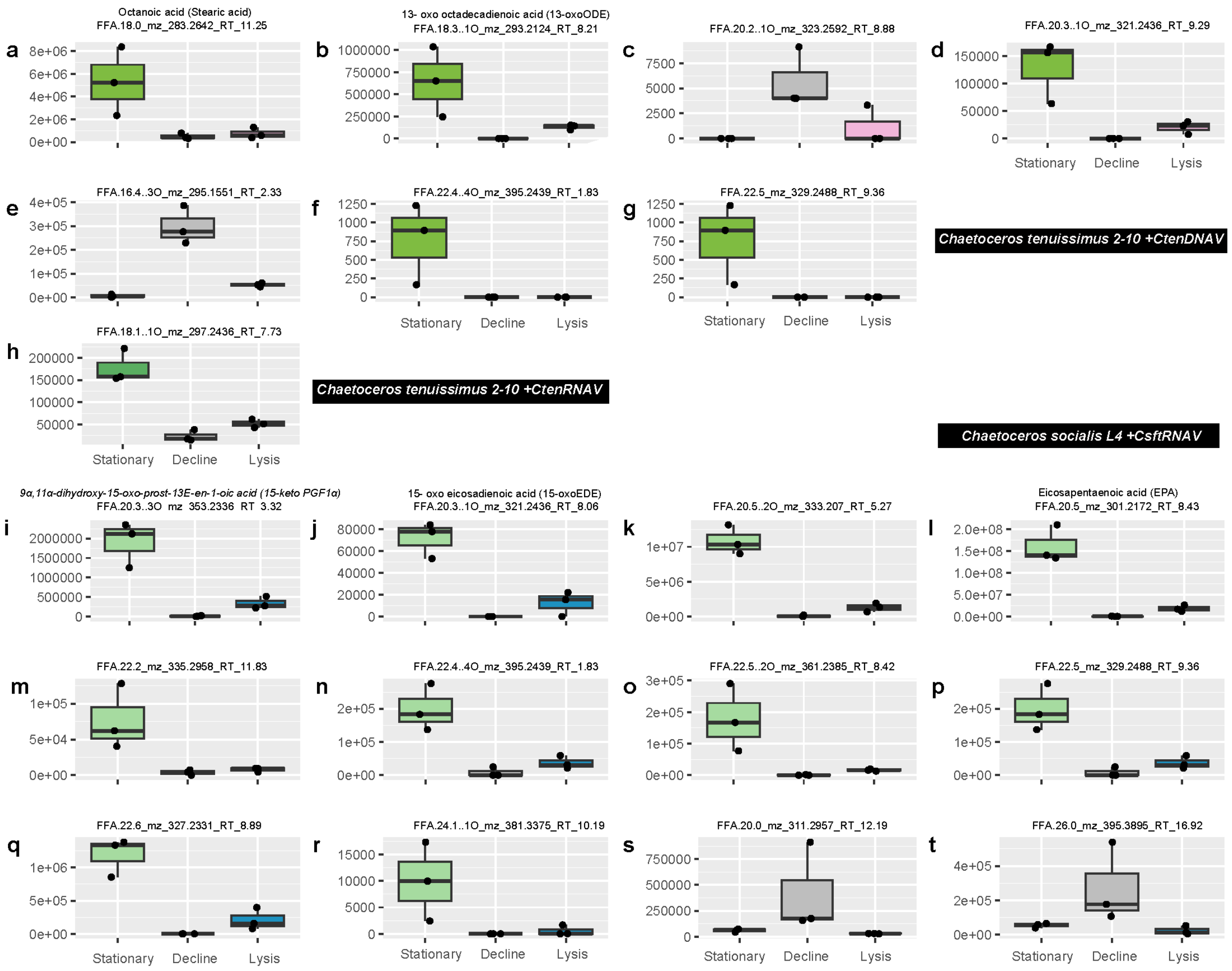

Figure S15. Box and whisker plots comparing compounds identified as being significantly more abundant in stationary (light greens) or decline phase (grey) control lipidomes relative to the lipidomes produced during viral lysis due to CtenDNAV (**a-g**; pink), CtenRNAV (**h**; lilac), and CsfrRNAV (**i-t;** blue). ANOVA, Fisher's post-hoc test FDR < 0.01.

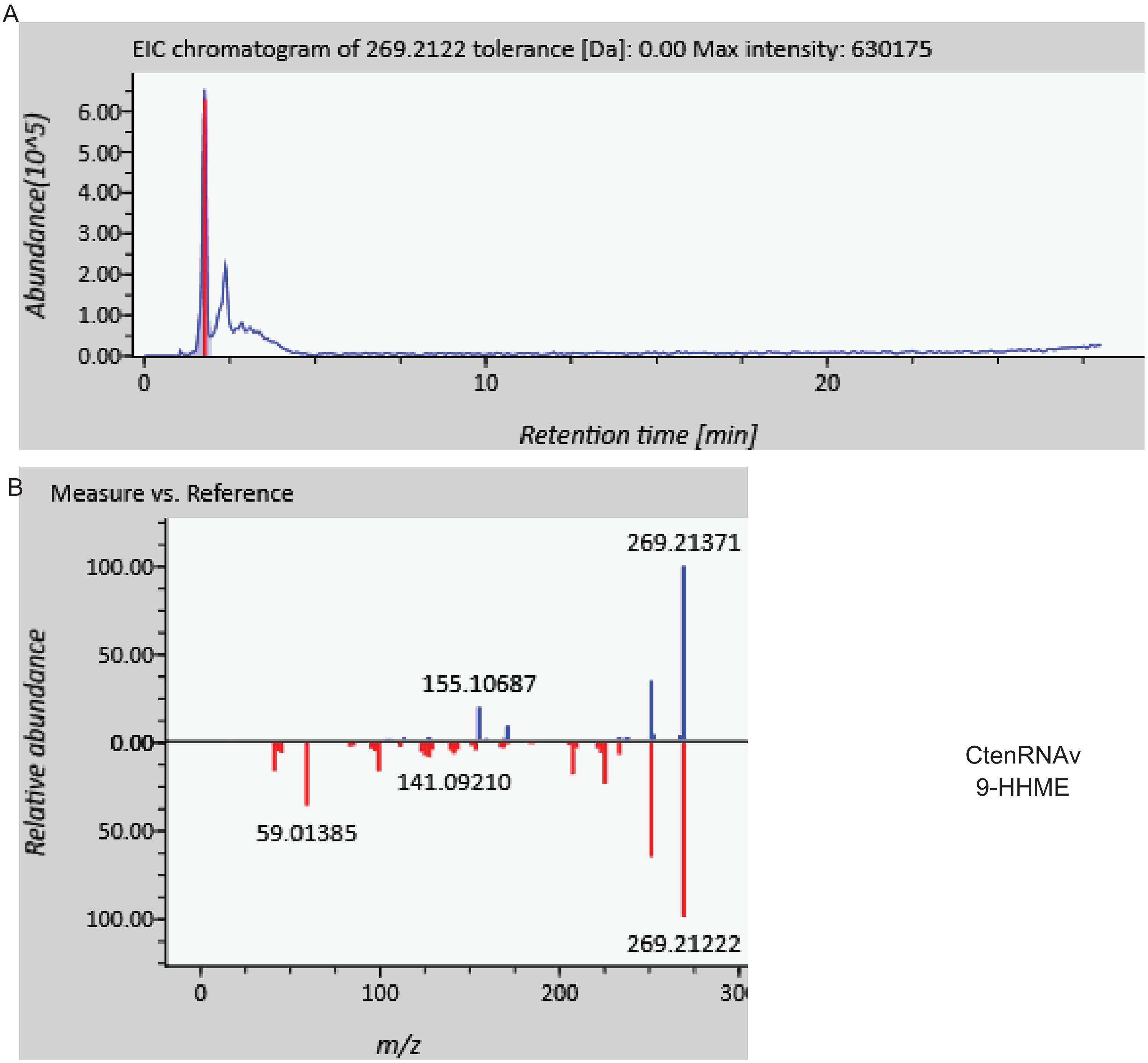

CtenRNAv
9-HHME

Figure S16) EIC and ms2 fragmentation for 9-HHME**. a)** Extracted ion chromatogram of the feature annotated as C16:1 oxylipin with 1 oxygen using exact mass and annotated to the positional level using ms2 fragmentation as 9-hydroxy hexadecaenoic acid (9-HHME) from the CtenRNAv experiment. **b)** Comparison of the observed fragmentation for m/z= 269.2123 at RT = 4.37 minutes to the fragmentation of the strongest match in a boutique database of modeled oxylipin fragmentation (Supplemental File 1A).

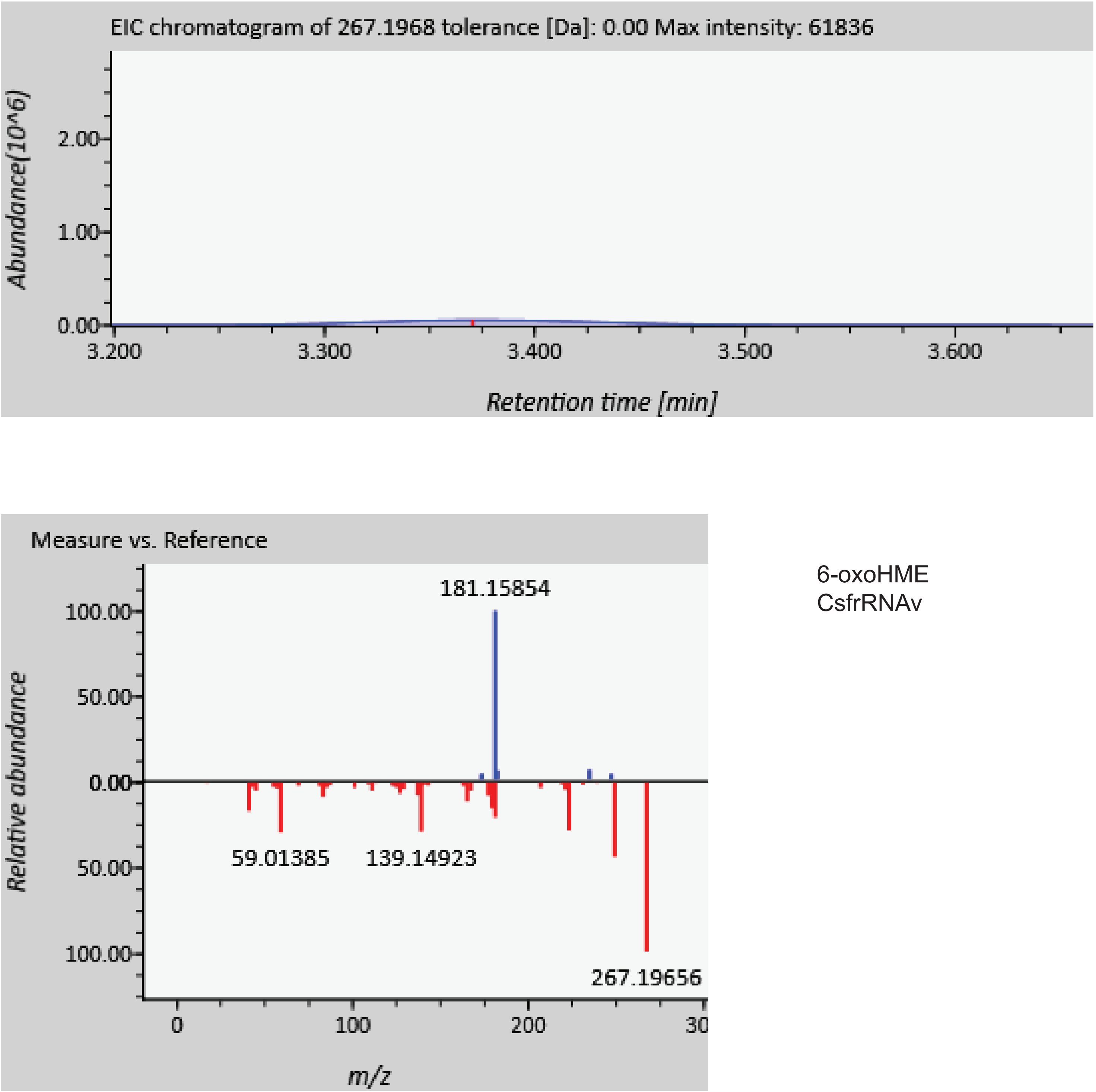

6-oxoHME
CsfrRNAv

Figure S17. EIC and ms2 fragmentation for 6-oxoHME **a)** Extracted ion chromatogram of the feature annotated as C16:2 oxylipin with 1 oxygen using exact mass and annotated to the positional level using ms2 fragmentation as 6-oxo hexadecaenoic acid (6-oxoHME) from the CsfrRNAv experiment. **b)** Comparison of the observed fragmentation for m/z= 267.1966 at RT = 3.46 minutes to the fragmentation of the strongest match in a boutique database of modeled oxylipin fragmentation (Supplemental File 1A).

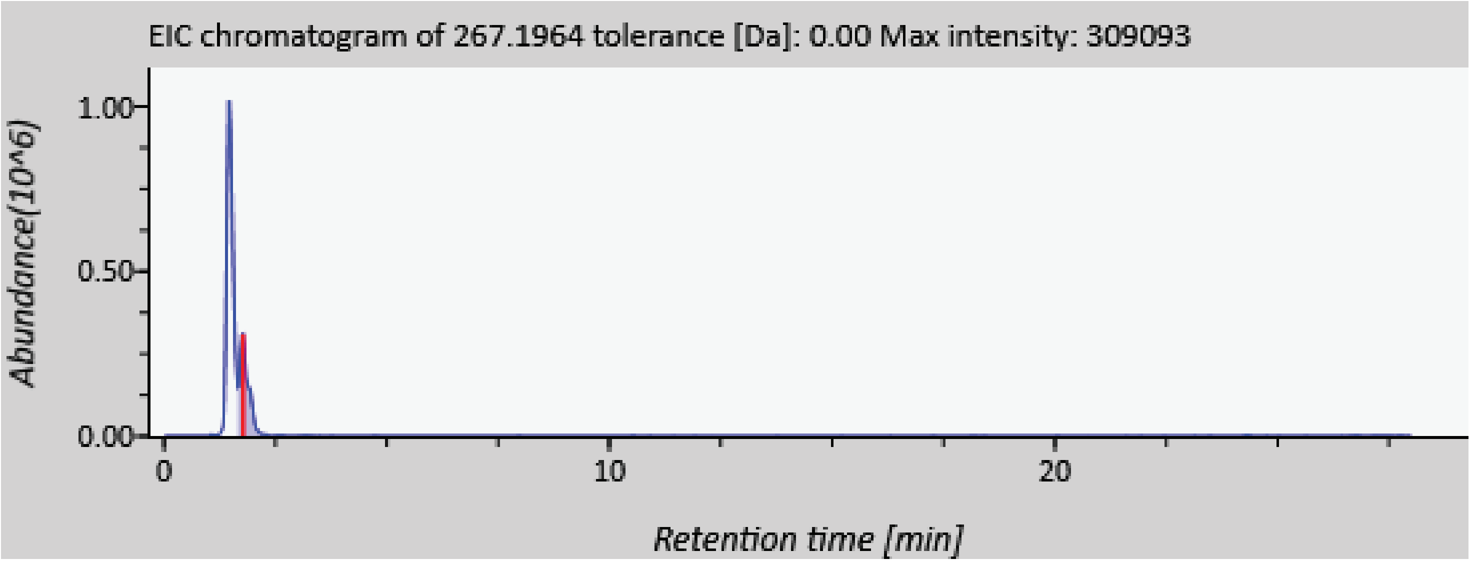

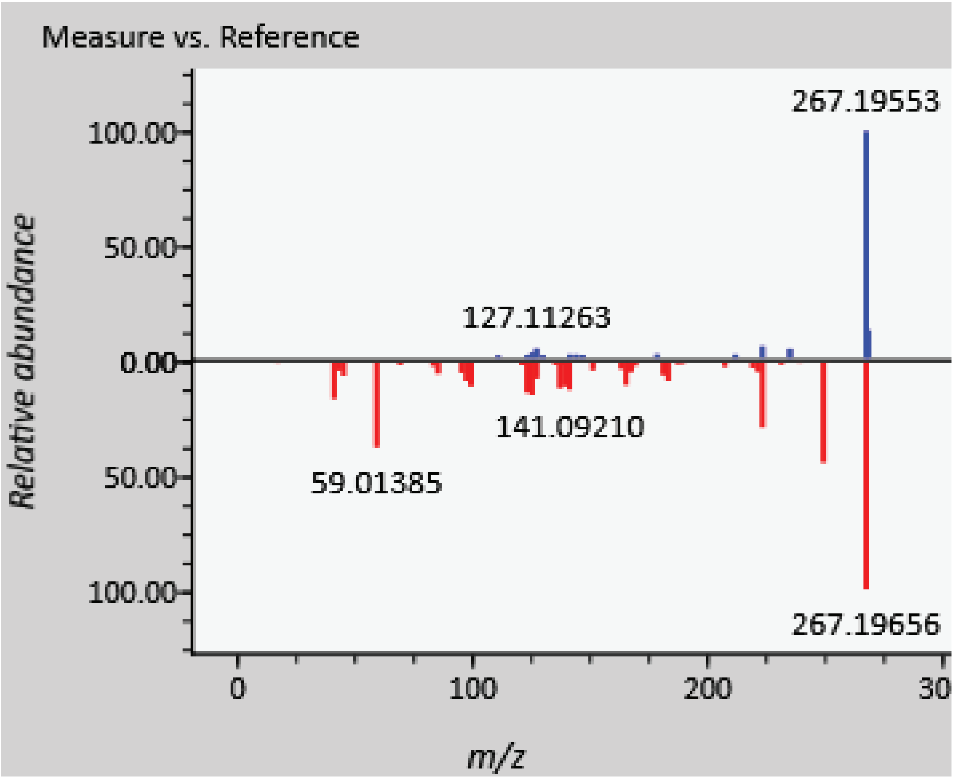

9-oxoHME
CtenRNAv

Figure S18. EIC and ms2 fragmentation for 9-oxoHME. **a)** Extracted ion chromatogram of the feature annotated as C16:2 oxylipin with1 oxygen using exact mass and annotated to the positional level using ms2 fragmentation as 9-oxo hexadecaenoic acid (9-oxoHME) from the CtenRNAv experiment. **b)** Comparison of the observed fragmentation for m/z= 267.1966 at RT = 4.78 minutes to the fragmentation of the strongest match in a boutique database of modeled oxylipin fragmentation (Supplemental File 1A).

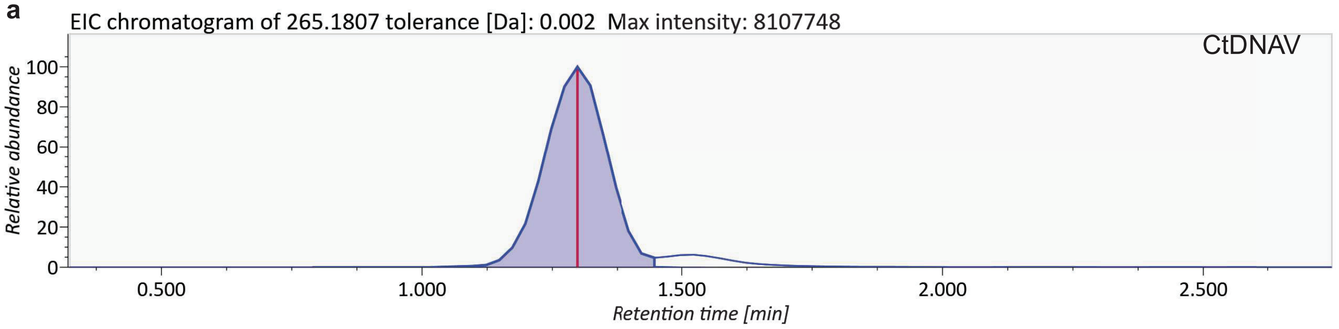

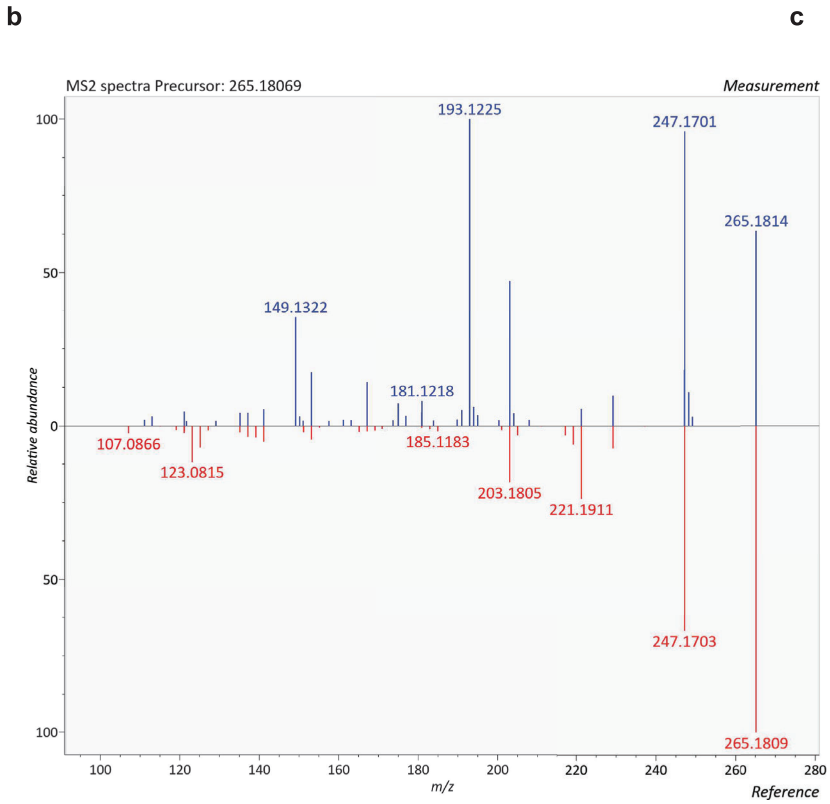

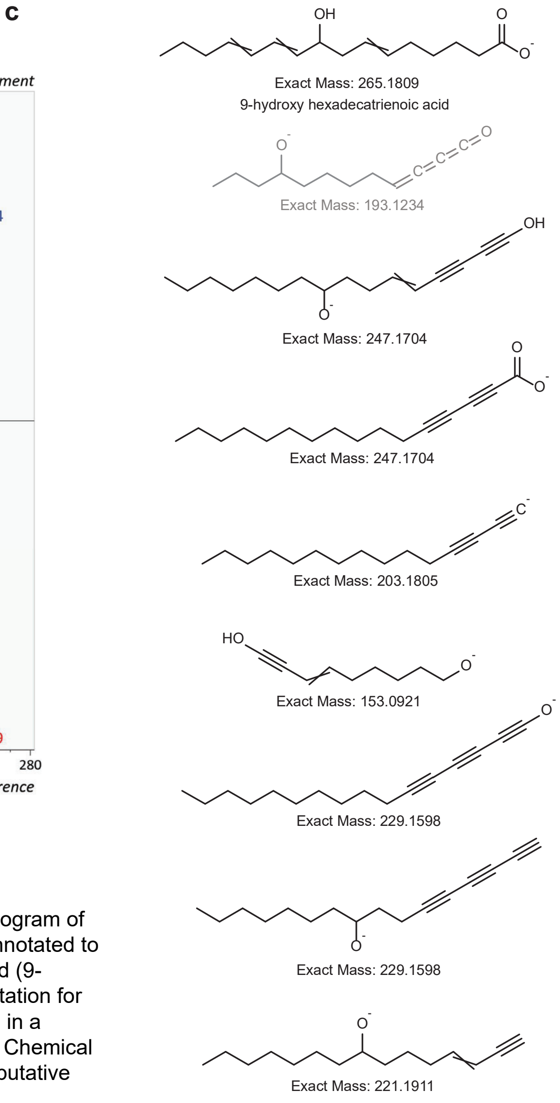

Figure S19. EIC and ms2 fragmentation for 9-HHTrE. **a)** Extracted ion chromatogram of the feature annotated as C16:3 oxylipin with 1 oxygen using exact mass and annotated to the positional level using ms2 fragmentation as 9-hydroxy hexadecatrienoic acid (9-HHTrE) from the CtDNAV experiment. **b)** Comparison of the observed fragmentation for m/z= 265.18069 at RT 2.23minutes to the fragmentation of the strongest match in a boutique database of modeled oxylipin fragmentation (Supplemental File 1). C) Chemical structure of the proposed parent Ion and fragments for the ms2 that match the putative annotation's modeled fragmentation.

a

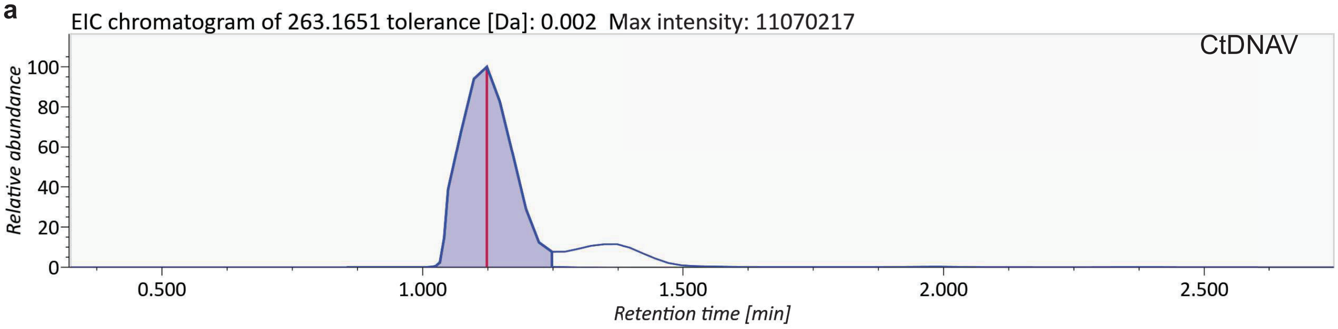

b

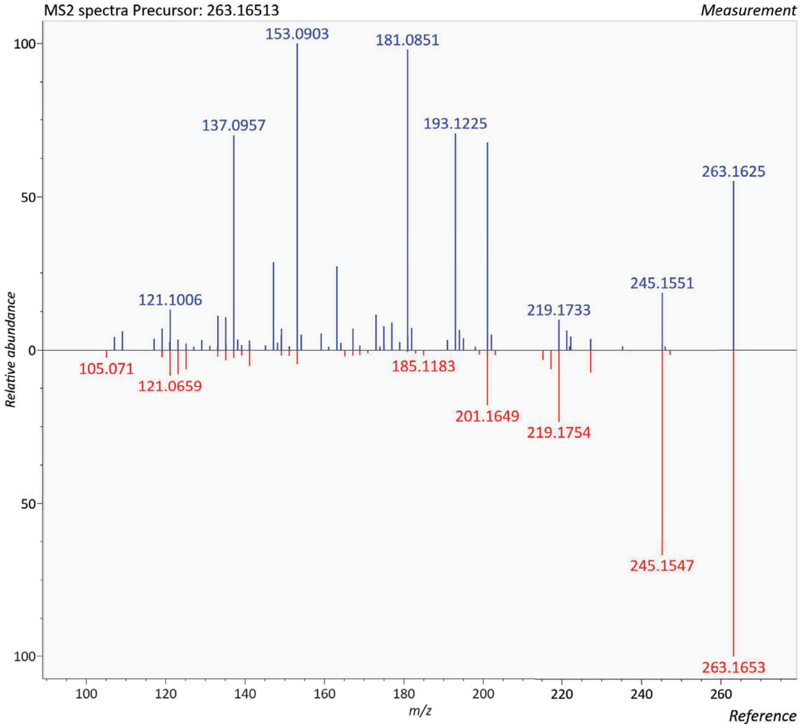

c

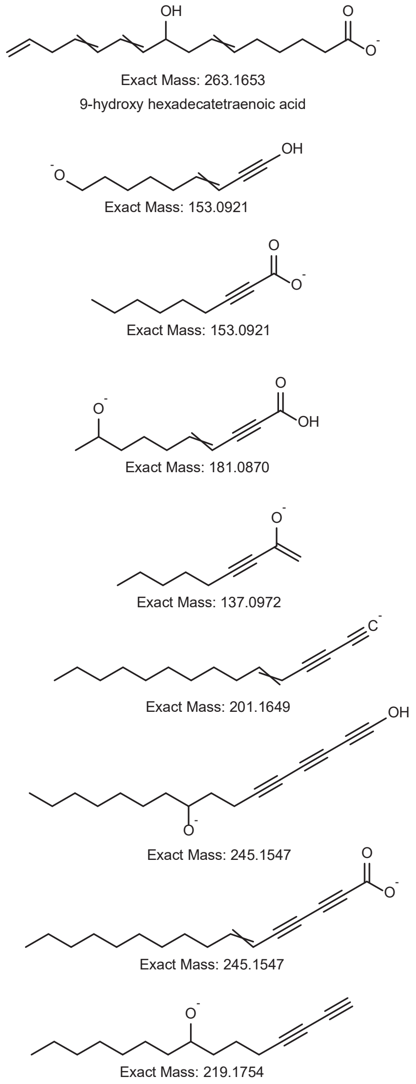

Figure S20. EIC and ms2 fragmentation for 9-HHTE. **a)** Extracted ion chromatogram of the feature annotated as C16:4 oxylipin with 1 oxygen using exact mass and annotated to the positional level using ms2 fragmentation as 9-hydroxy hexadecatetraenoic acid (9-HHTE) from the CtDNAv experiment. **b)** Comparison of the observed fragmentation for m/z= 263.1651 at RT = 2.46 minutes to the fragmentation of the strongest match in a boutique database of modeled oxylipin fragmentation (Supplemental File 1A). C) Chemical structure of the proposed parent Ion and fragments for the ms2 that match the putative annotation's modeled fragmentation.

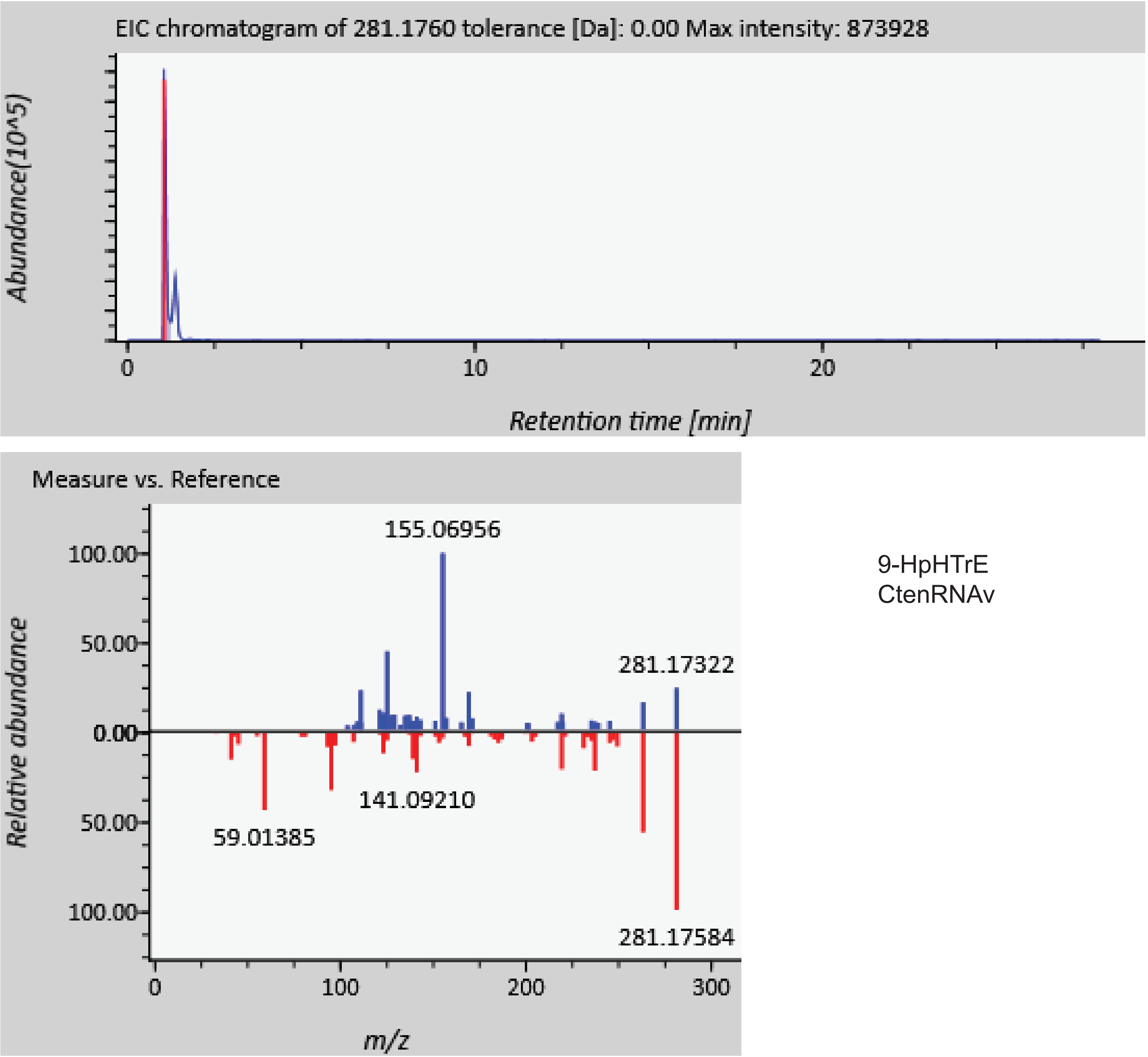

9-HpHTrE
CtenRNAv

Figure S21. EIC and ms2 fragmentation for 9-HpHTE. **a)** Extracted ion chromatogram of the feature annotated as C16:3 oxylipin with 2 oxygens using exact mass and annotated to the positional level using ms2 fragmentation as 9-hydroperoxy hexadecatrienoic acid (9-HpHTrE) from the CtenRNAv experiment. **b)** Comparison of the observed fragmentation for m/z= 281.1759 at RT = 2.23 minutes to the fragmentation of the strongest match in a boutique database of modeled oxylipin fragmentation (Supplemental File 1A).

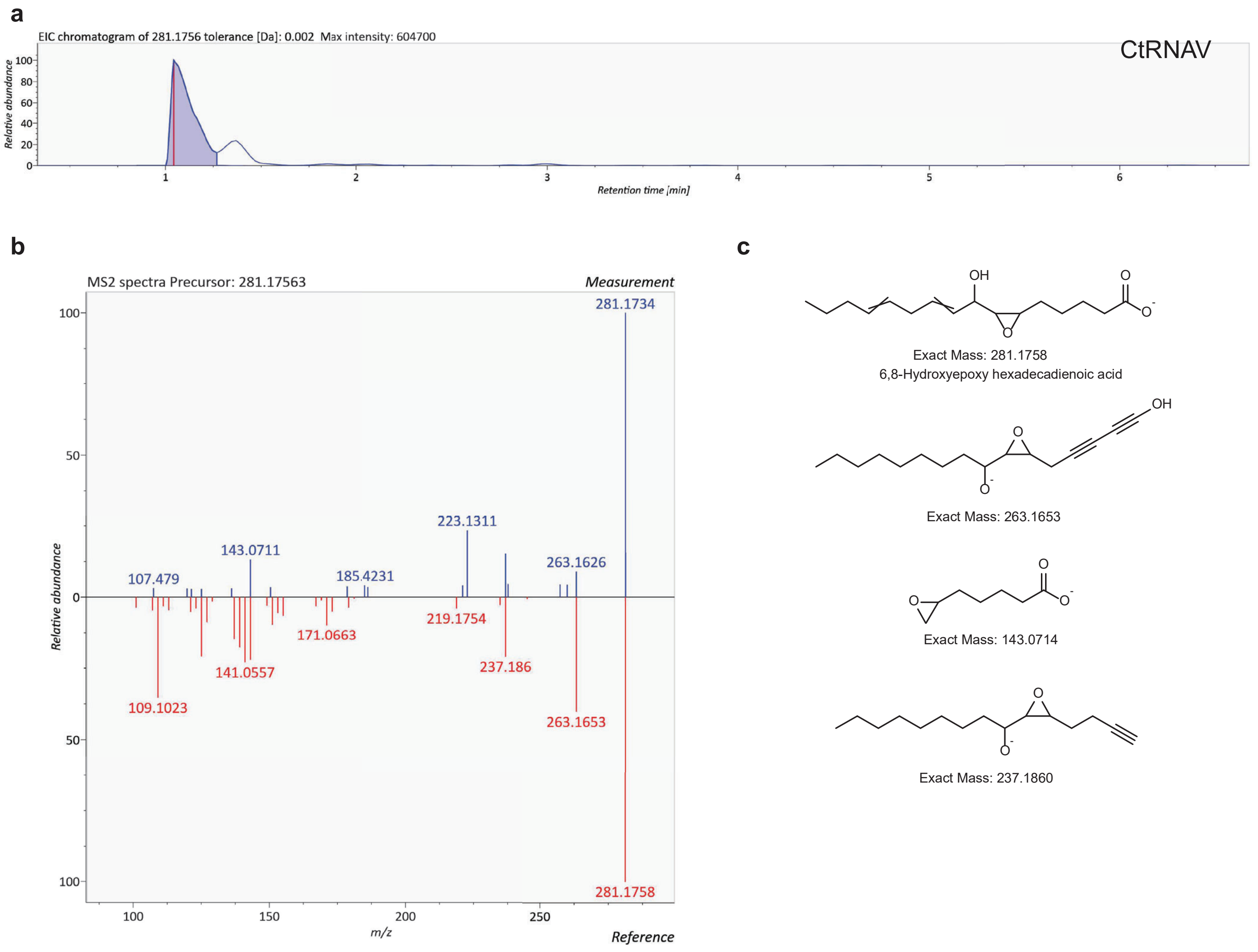

Figure S22. EIC and ms2 fragmentation for 6,8-HepHDE. **a)** Extracted ion chromatogram of the feature annotated as C16:3 oxylipin with 2 oxygens using exact mass and annotated to the positional level using ms2 fragmentation as 6,8-hydroxyepoxy hexadecadienoic acid (6,8-HepHDE) from the CtenRNAv experiment. **b)** Comparison of the observed fragmentation for m/z= 281.1759 at RT = 3.21 minutes to the fragmentation of the strongest match in a boutique database of modeled oxylipin fragmentation (Supplemental File 1A). **c)** chemical structures of ms2 matching the CFM-ID database.

a

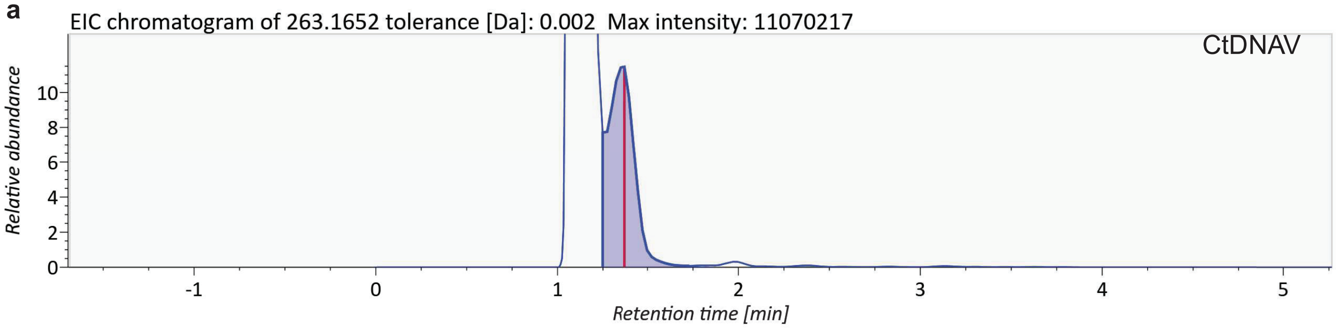

b

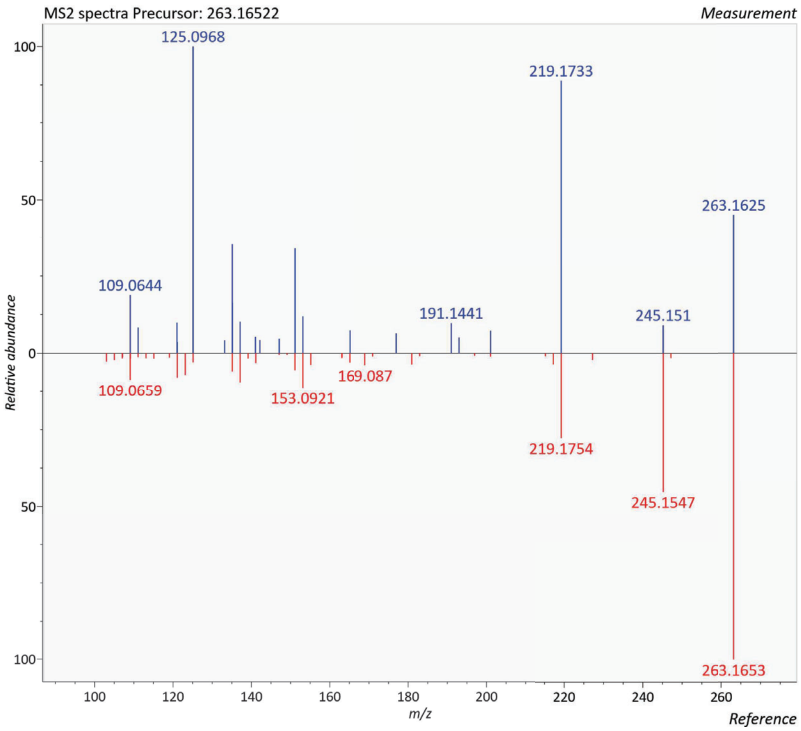

c

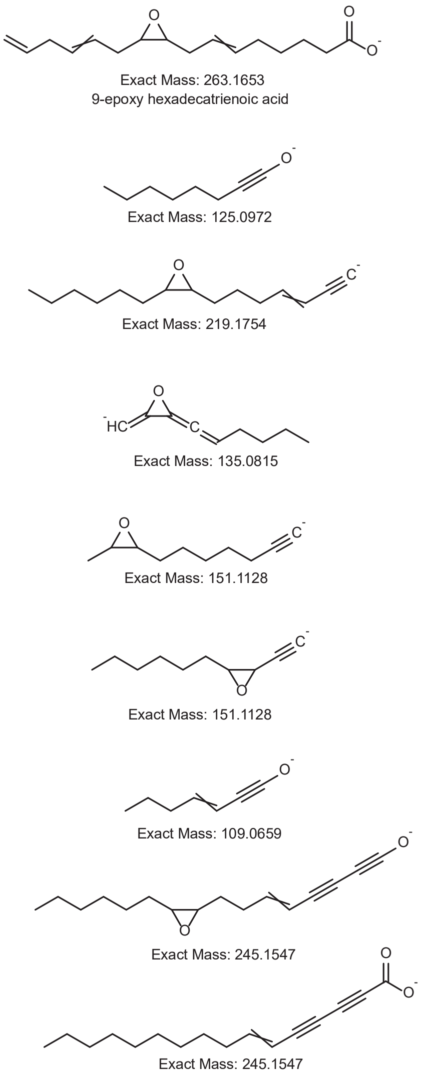

Figure S23. EIC and ms2 fragmentation for 9-epHTrE. **a)** Extracted ion chromatogram of the feature annotated as C16:4 oxylipin with 1 oxygen using exact mass and annotated to the positional level using ms2 fragmentation as 9-epoxy hexadecatrienoic acid (9-epHTrE) from the CtDNAv experiment. **b)** Comparison of the observed fragmentation for m/z= 263.16522 at RT =3.03 minutes to the fragmentation of the strongest match in a boutique database of modeled oxylipin fragmentation (Supplemental File 1A). **c)** Chemical structure of the proposed parent Ion and fragments for the ms2 that match the putative annotation's modeled fragmentation.

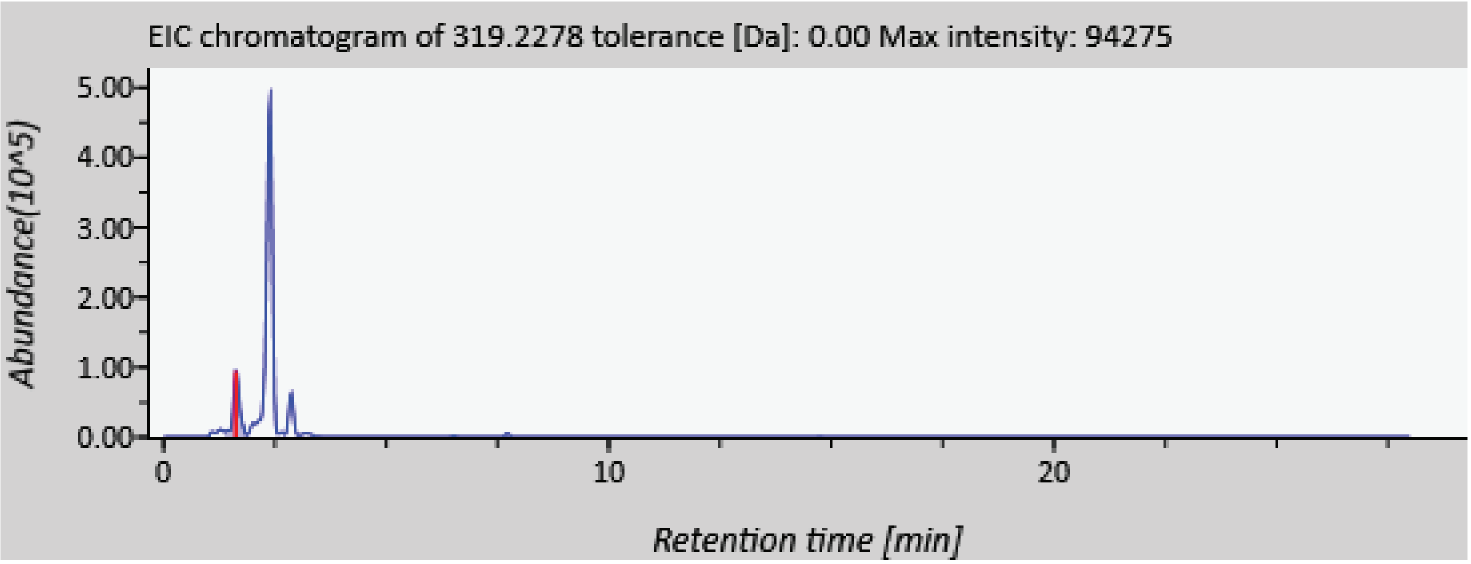

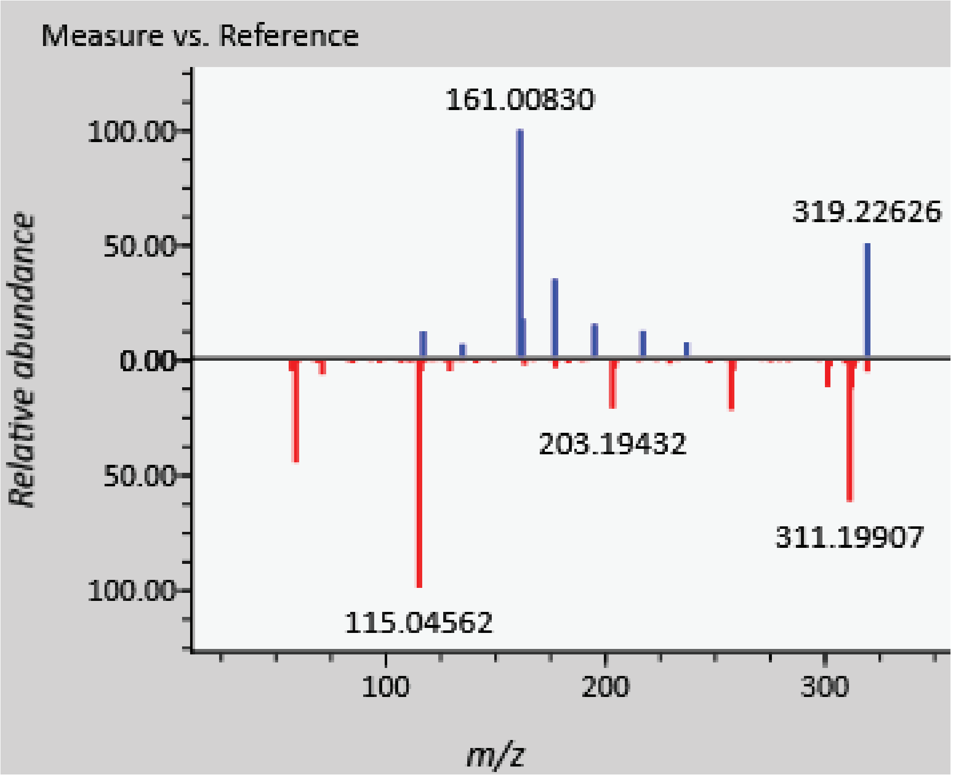

8-HETE
CtenRNAv

Figure S24. EIC and ms2 fragmentation for 8-HETE. **a**) Extracted ion chromatogram of the feature annotated as C20:4 oxylipin with 1 oxygen using exact mass and annotated to the positional level using ms2 fragmentation as 8-hyrdoxy eicosatetraenoic acid (8-HETE) from the CtenRNAv experiment. b) Comparison of the observed fragmentation for m/z= 319.2275 at RT = 4.57 minutes to the fragmentation of the strongest match in a boutique database of modeled oxylipin fragmentation (Supplemental File 1A).

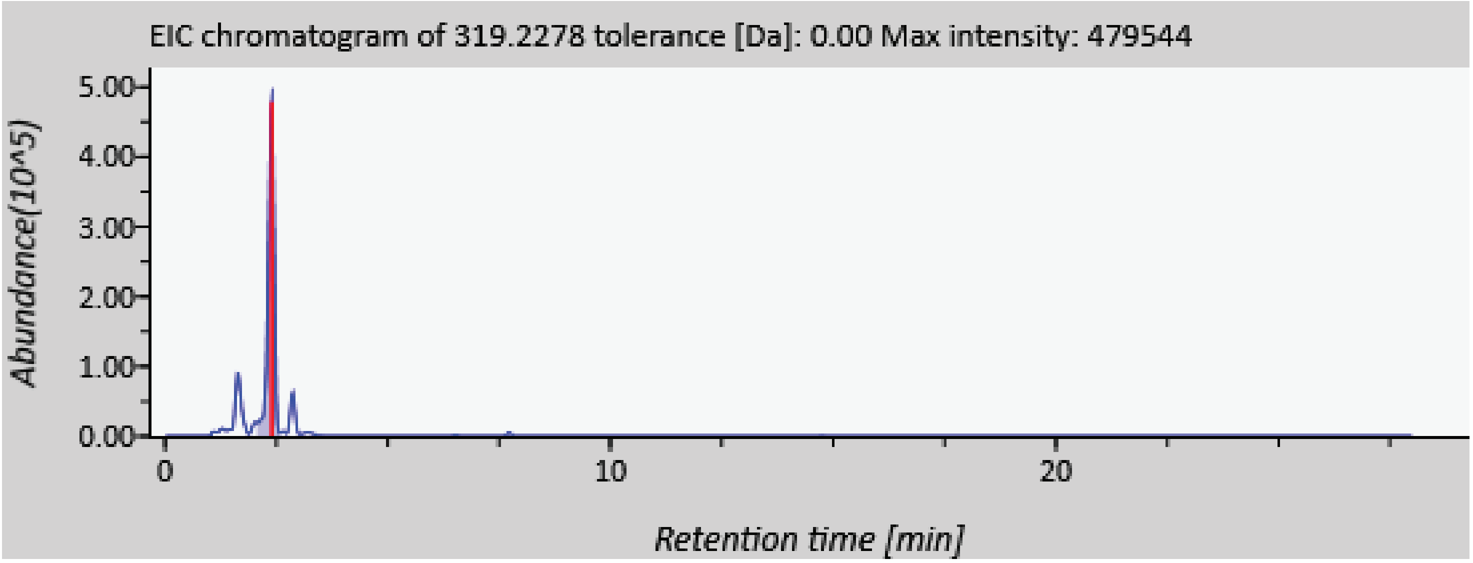

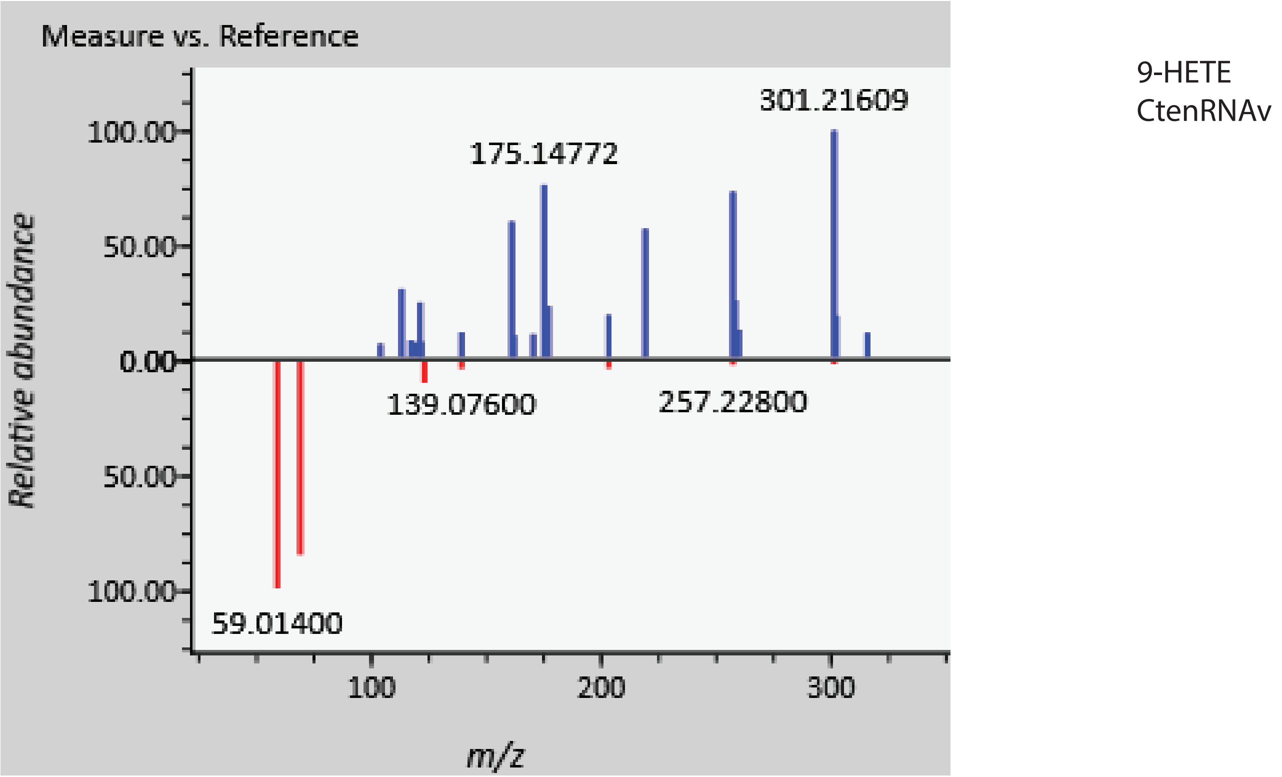

Figure S25. EIC and ms2 fragmentation for 9-HETE. **a)** Extracted ion chromatogram of the feature annotated as C20:4 oxylipin with 1 oxygen using exact mass and annotated to the positional level using ms2 fragmentation as 8-hyrdoxy eicosatetraenoic acid (9-HETE) from the CtenRNAv experiment. **b)** Comparison of the observed fragmentation for m/z= 319.2275 at RT = 5.95 minutes to the fragmentation of the strongest match in a boutique database of modeled oxylipin fragmentation (Supplemental File 1A).

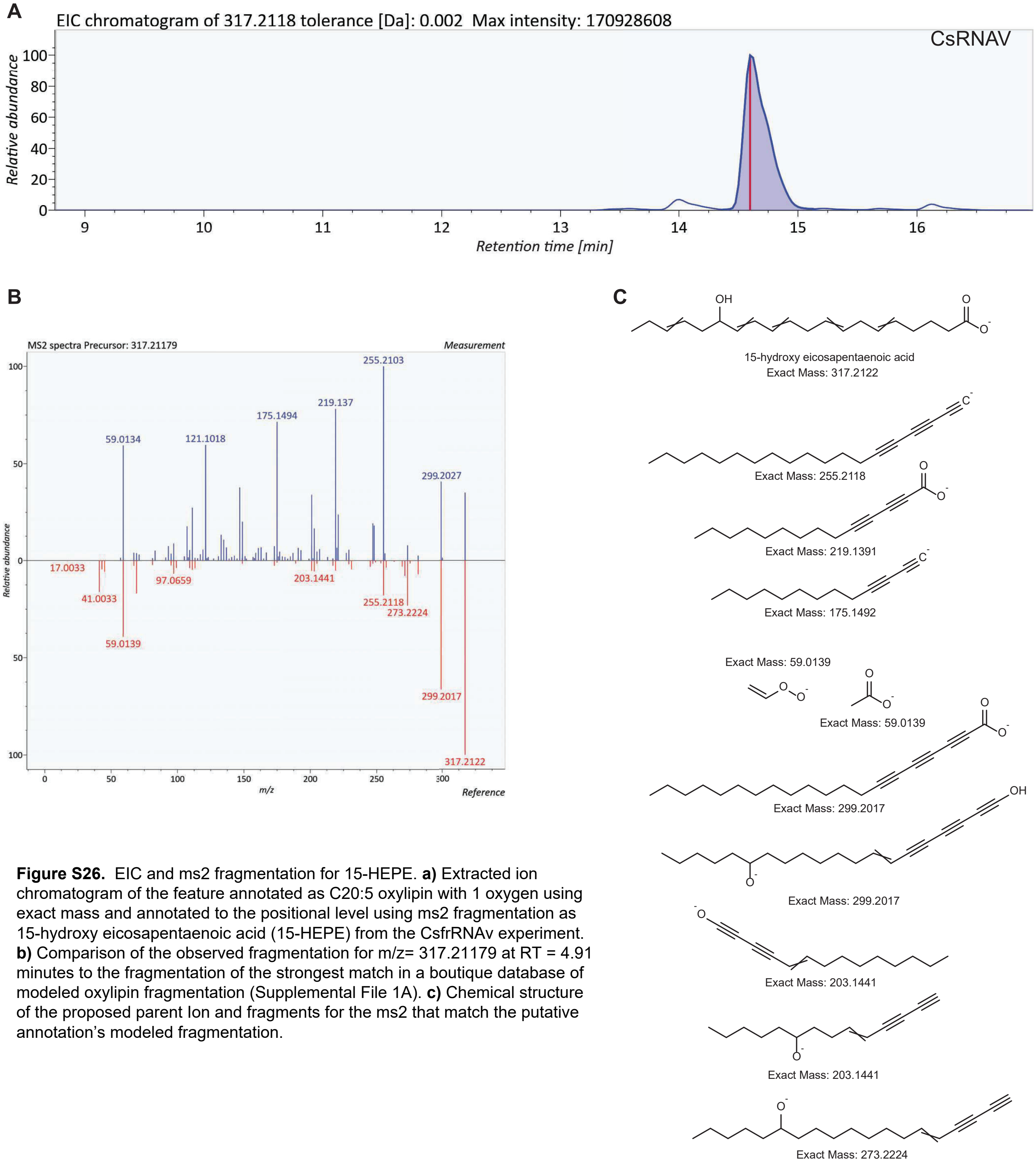

Figure S26. EIC and ms2 fragmentation for 15-HEPE. **a)** Extracted ion chromatogram of the feature annotated as C20:5 oxylipin with 1 oxygen using exact mass and annotated to the positional level using ms2 fragmentation as 15-hydroxy eicosapentaenoic acid (15-HEPE) from the CsfrRNAv experiment. **b)** Comparison of the observed fragmentation for m/z= 317.21179 at RT = 4.91 minutes to the fragmentation of the strongest match in a boutique database of modeled oxylipin fragmentation (Supplemental File 1A). **c)** Chemical structure of the proposed parent Ion and fragments for the ms2 that match the putative annotation's modeled fragmentation.

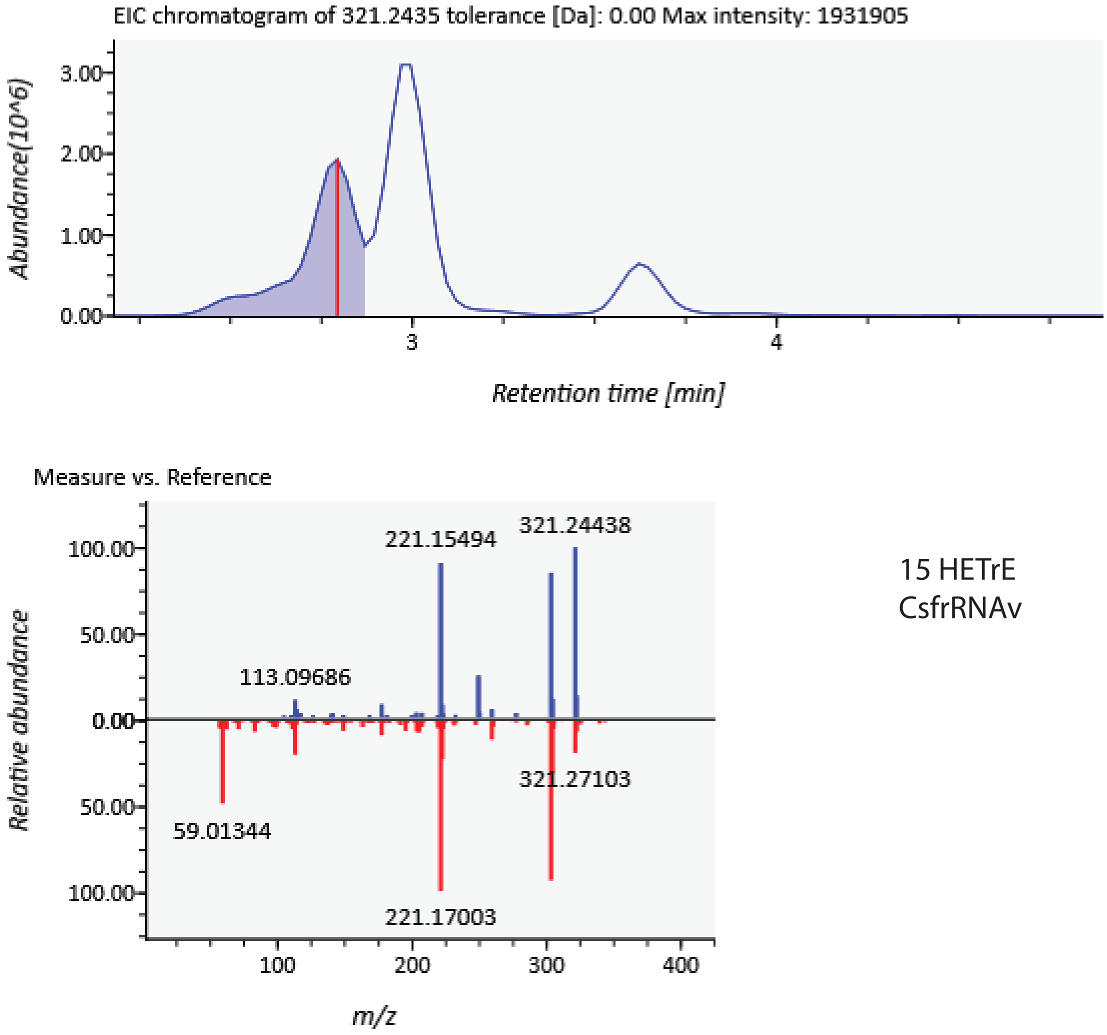

Figure S27. EIC and ms2 fragmentation for 15-HETrE. **a)** Extracted ion chromatogram of the feature annotated as C20:3 oxylipin with 1 oxygen using exact mass and annotated to the positional level using ms2 fragmentation as 15-hydroxy eicosatrienoic acid (15-HETrE) from the CsRNAv experiment. **b)** Comparison of the observed fragmentation for m/z= 321.2435 at RT = 6.98 minutes to the fragmentation of the strongest match in a boutique database of modeled oxylipin fragmentation (Supplemental File 1A).

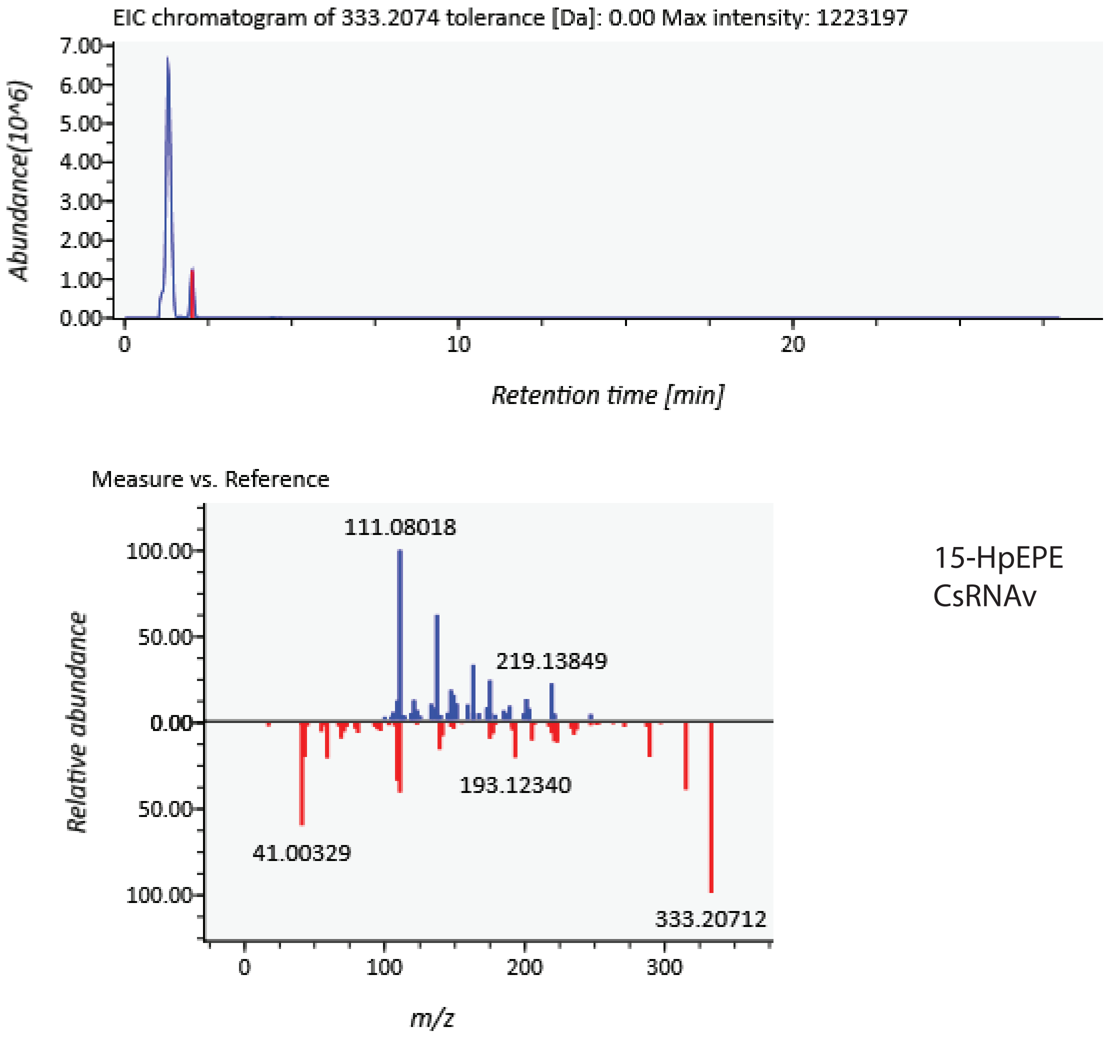

Figure S28. EIC and ms2 fragmentation for 15-HpEPE. **a)** Extracted ion chromatogram of the feature annotated as C20:5 oxylipin with 2 oxygens using exact mass and annotated to the positional level using ms2 fragmentation as 15-hydroperoxy eicosapentaenoic acid (15-HpEPE) from the CsfrRNAv experiment. **b)** Comparison of the observed fragmentation for m/z= 333.207 at RT =5.27 minutes to the fragmentation of the strongest match in a boutique database of modeled oxylipin fragmentation (Supplemental File 1A).

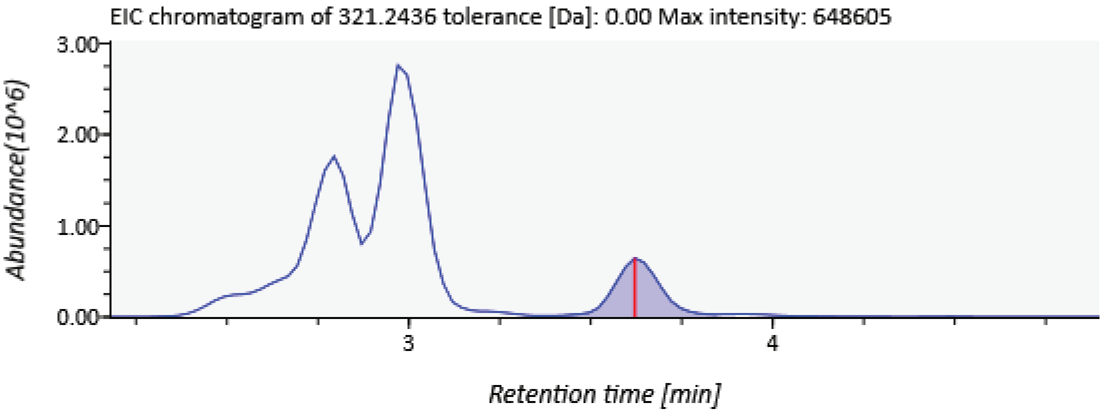

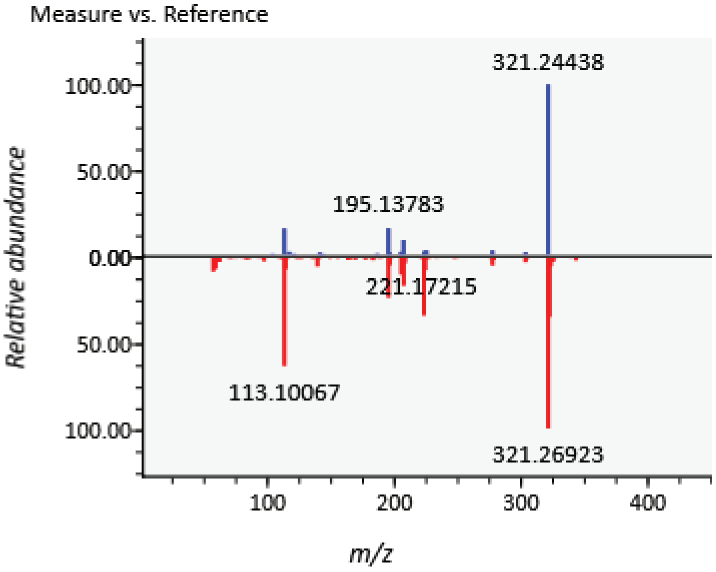

15-oxoHDE
CsRNAv
*ms2 precision a little off so
Dot Prod, reverse prod, and
presence scores weren't calculated

Figure S29. EIC and ms2 fragmentation for 15-oxoHDE. **a)** Extracted ion chromatogram of the feature annotated as C20:3 oxylipin with 1 oxygen using exact mass and annotated to the positional level using ms2 fragmentation as 15-oxo eicosadienoic acid 15-oxoEDE) from the CsfrRNAv experiment. **b)** Comparison of the observed fragmentation for m/z= 321.2436 at RT = 8.06 minutes to the fragmentation of the strongest match in a boutique database of modeled oxylipin fragmentation (Supplemental File 1A).

a

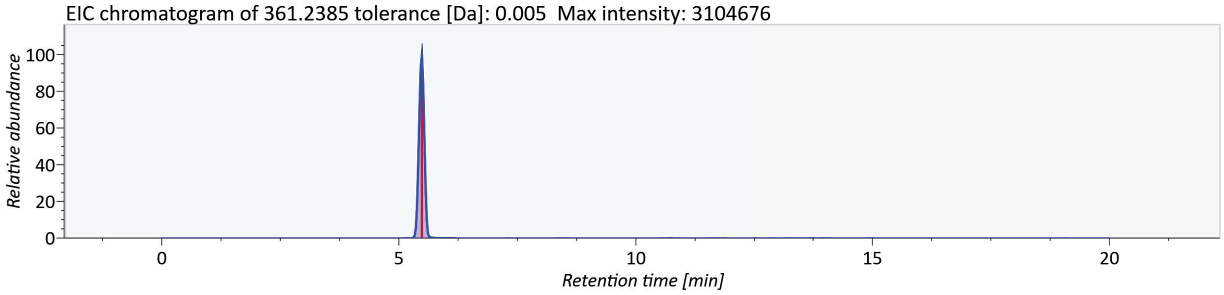

b

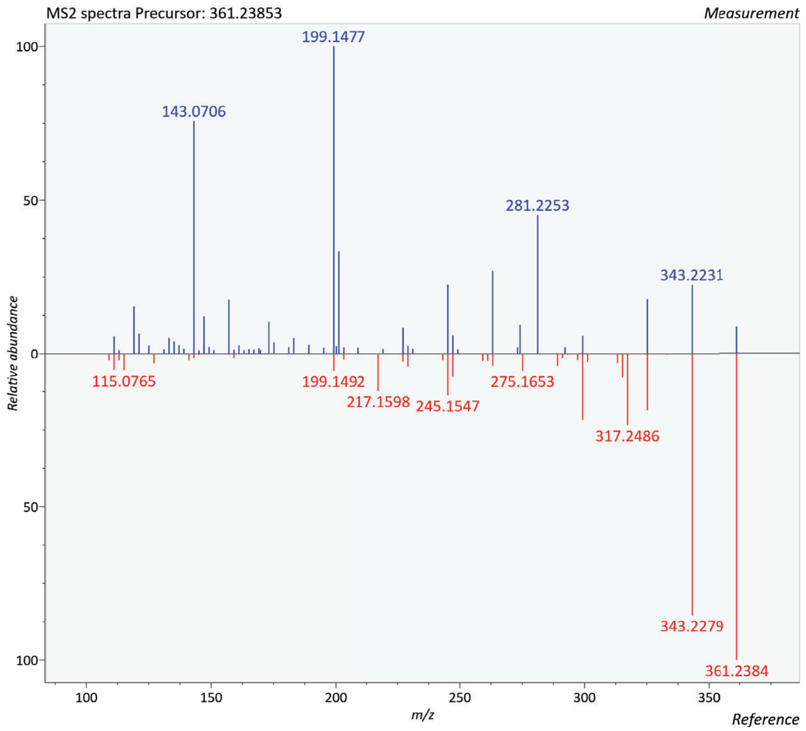

c

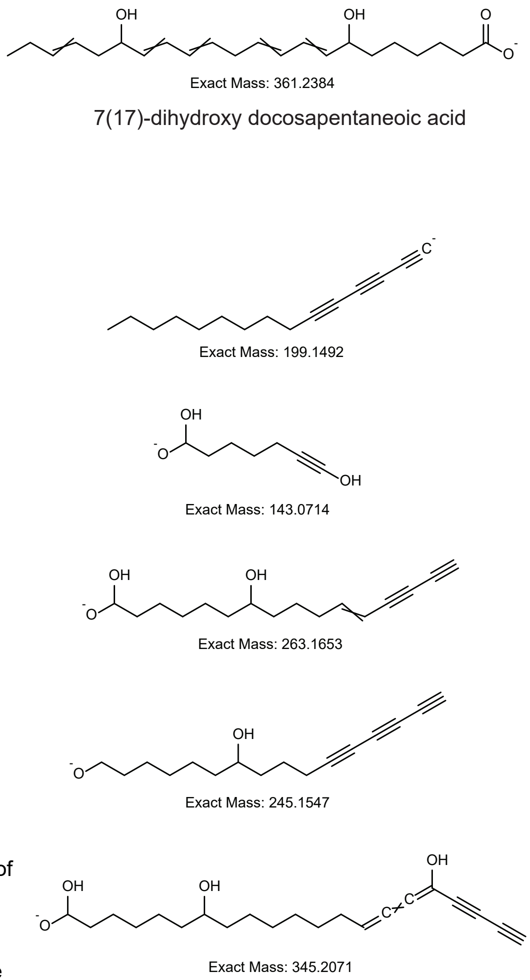

Figure S30. EIC and ms2 fragmentation for 7,17-diHDPA. **a)** Extracted ion chromatogram of the 7(17) dihydroxy docosapentaenoic acid authentic standard eluting at 5.49 minutes. **b)** Comparison of the observed fragmentation for m/z= 361.2385 at RT = 5.49 minutes to the oxylipin fragmentation predicted using the CFMID package (Supplemental File 1C). A table quantifying the quality of the ms2 fragmentation match can be found in Supplemental Table 8. **c)** Chemical structure of the proposed parent ion and ms2 fragments that match the authentic standard's modeled fragmentation.

a

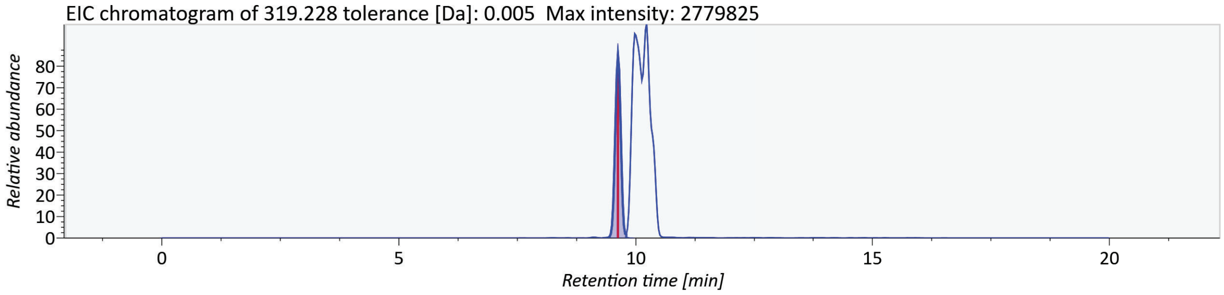

b

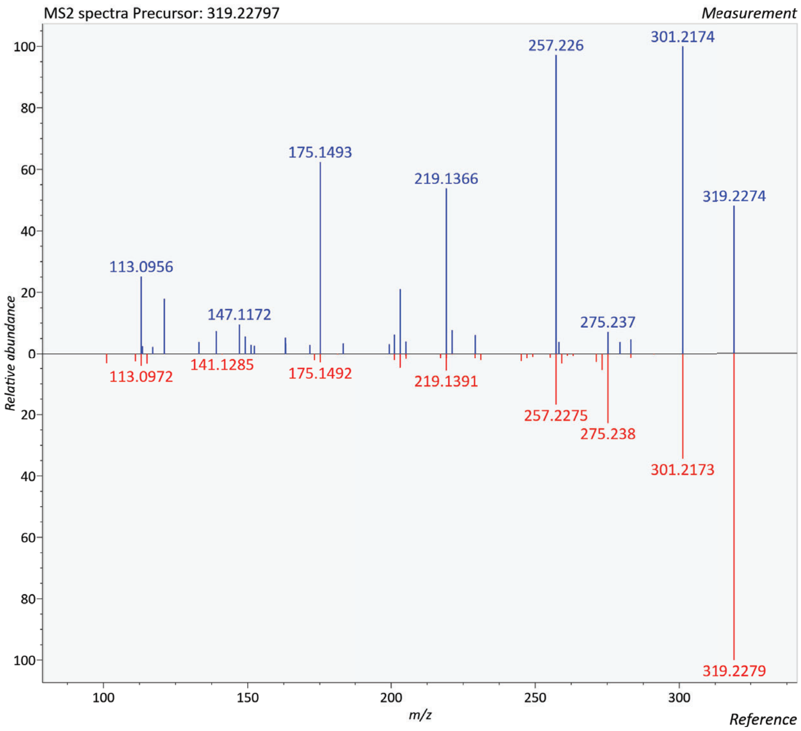

c

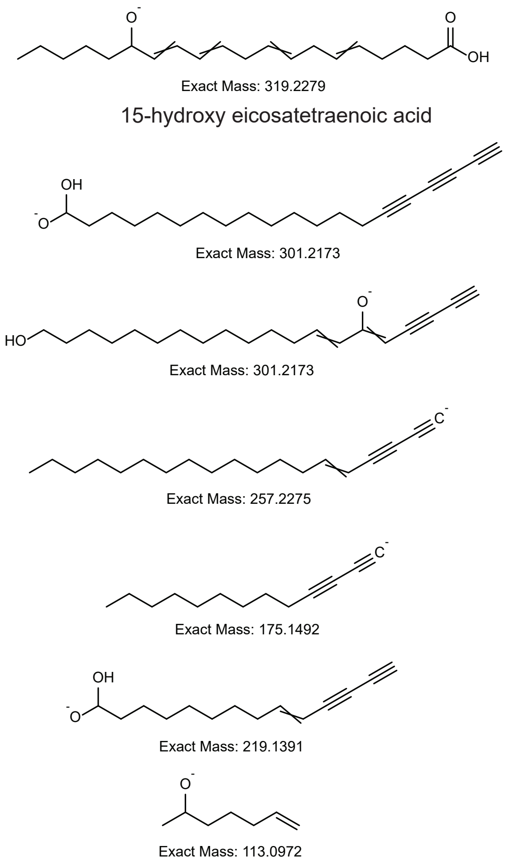

Figure S31. EIC and ms2 fragmentation for 15-HETE. **a)** Extracted ion chromatogram of the 15-hydroxy eicosatetraenoic acid authentic standard eluting at 9.6 min. **b)** Comparison of the observed fragmentation for m/z= 319.228 at RT = 9.6 minutes to the oxylipin fragmentation predicted using the CFMID package
(Supplemental File 1C). A table quantifying the quality of the ms2 fragmentation match can be found in Supplemental Table 8. **c)** Chemical structure of the proposed parent ion and ms2 fragments that match the authentic standard's modeled fragmentation.

a

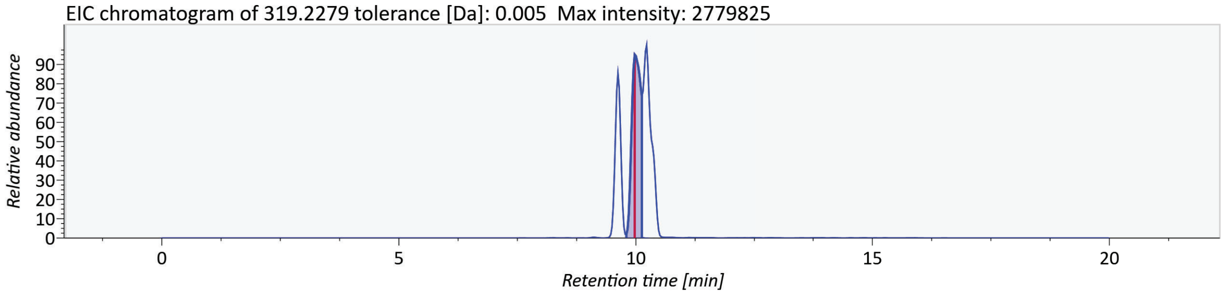

b

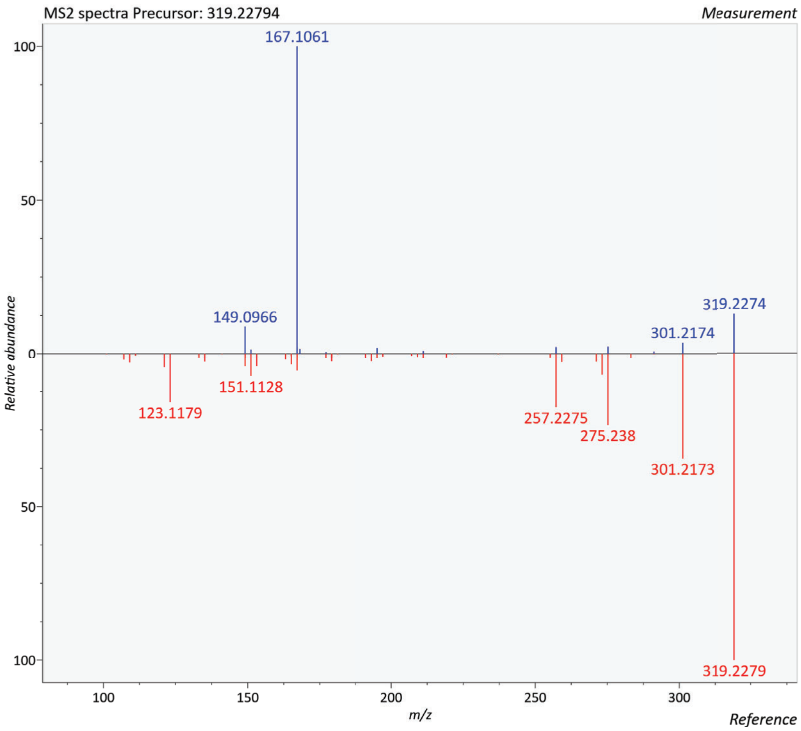

c

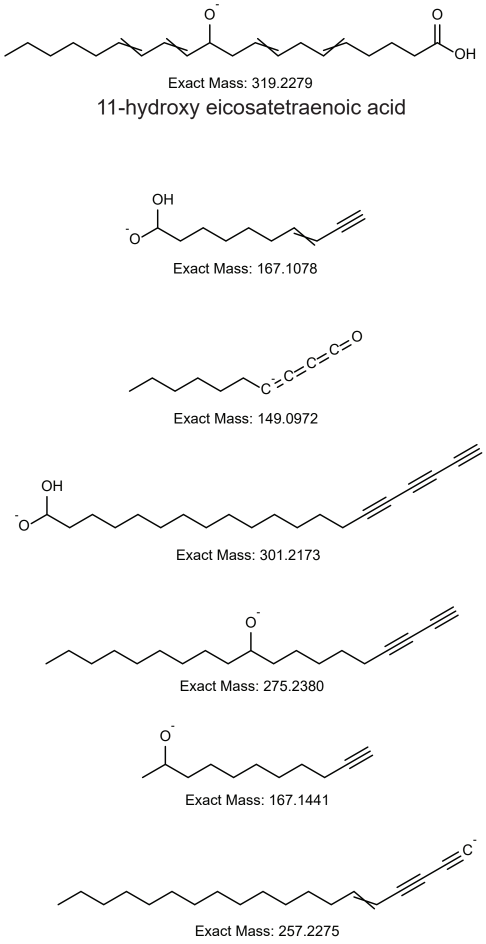

Figure S32. EIC and ms2 fragmentation for 11-HETE. **a)** Extracted ion chromatogram of the 11-hydroxy eicosatetraenoic acid authentic standard eluting at 9.9 min. **b)** Comparison of the observed fragmentation for m/z= 319.228 at RT = 9.9 minutes to the oxylipin fragmentation predicted using the CFMID package (Supplemental File 1C). A table quantifying the quality of the ms2 fragmentation match can be found in Supplemental Table 8. **c)** Chemical structure of the proposed parent ion and ms2 fragments that match the authentic standard's modeled fragmentation.

a

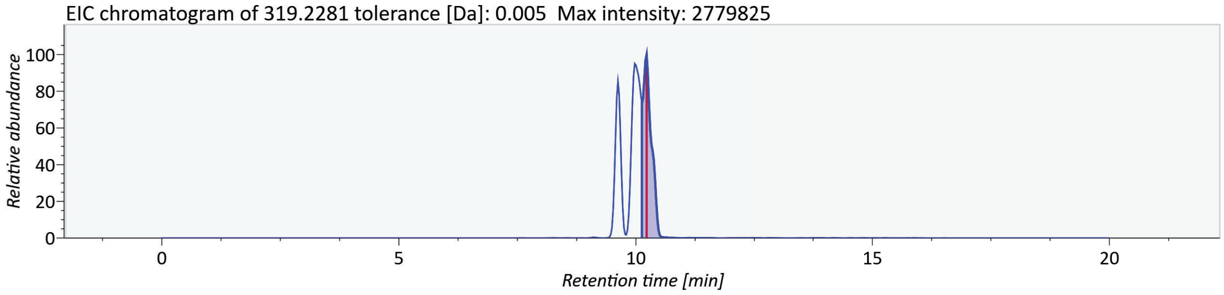

b

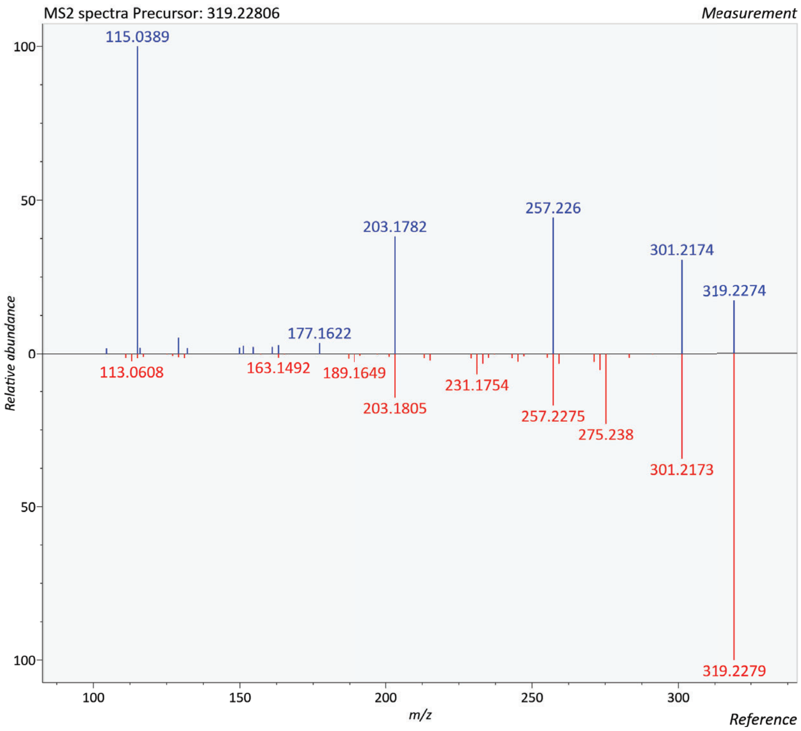

c

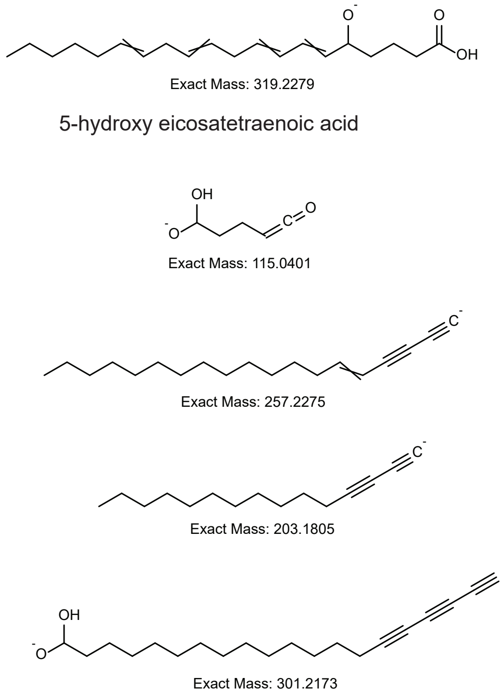

Figure S33. EIC and ms2 fragmentation for 5-HETE. **a)** Extracted ion chromatogram of the 5-hydroxy eicosatetraenoic acid authentic standard eluting at 10.2 min. **b)** Comparison of the observed fragmentation for m/z= 319.228 at RT = 10.2 minutes to the oxylipin fragmentation predicted using the CFMID package (Supplemental File 1C). A table quantifying the quality of the ms2 fragmentation match can be found in Supplemental Table 8. **c)** Chemical structure of the proposed parent ion and ms2 fragments that match the authentic standard's modeled fragmentation.

a

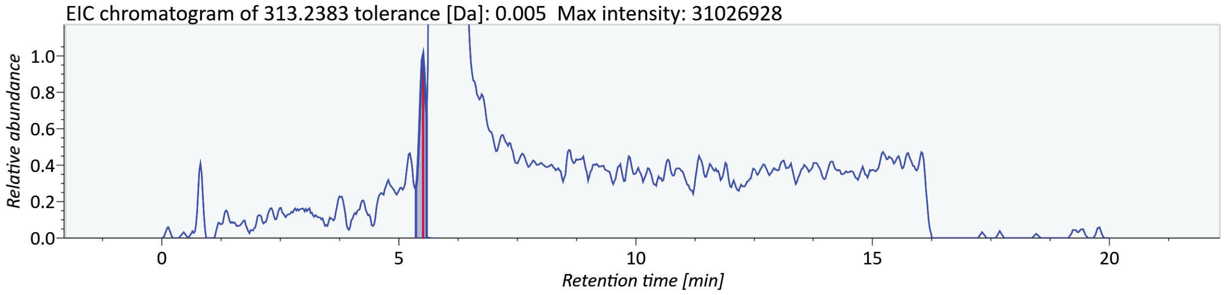

b

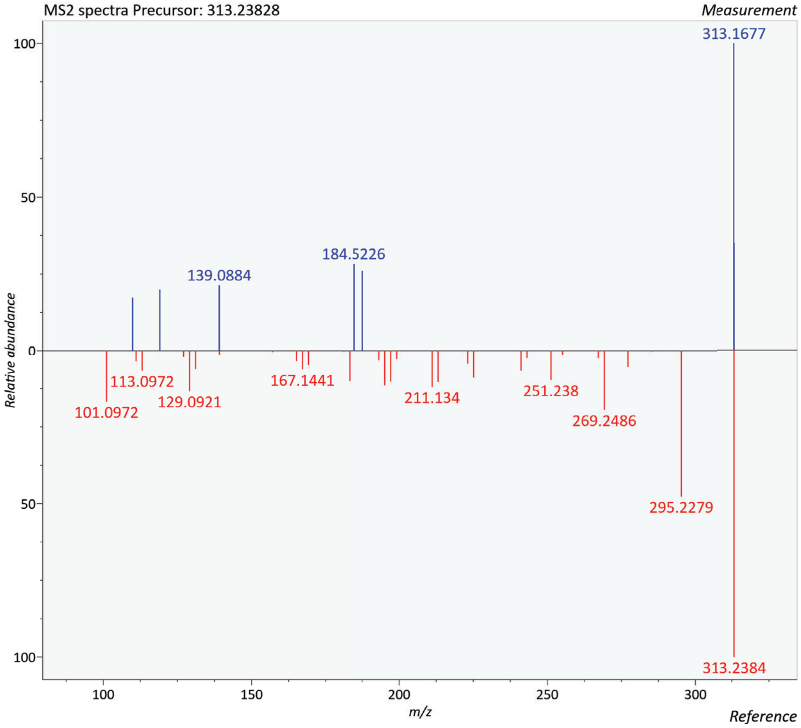

c

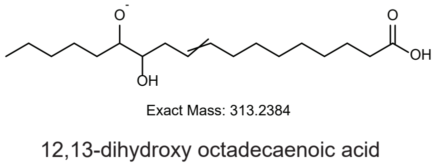

Figure S34. EIC and ms2 fragmentation for 12,13-diHODE. **a)** Extracted ion chromatogram of the 12,13-dihydroxy octadecaenoic acid authentic standard eluting at 5.5 min. **b)** Comparison of the observed fragmentation for m/z= 313.2385 at RT = 5.5 minutes to the oxylipin fragmentation predicted using the CFMID package (Supplemental File 1C). A table quantifying the quality of the ms2 fragmentation match can be found in Supplemental Table 8. **c)** Chemical structure of the proposed parent ion and ms2 fragments that match the authentic standard's modeled fragmentation.

a

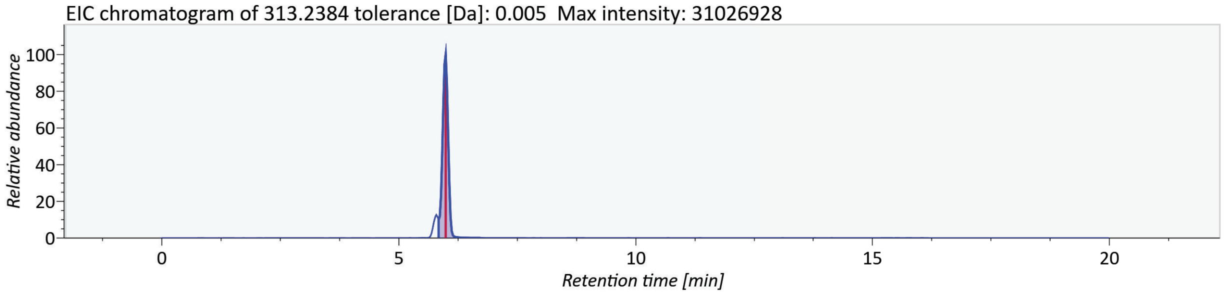

b

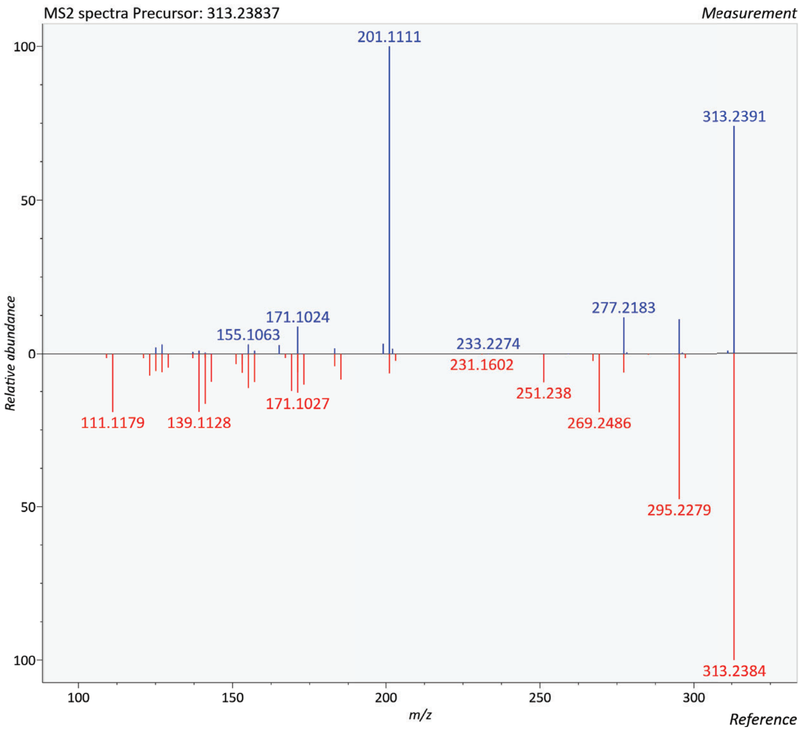

c

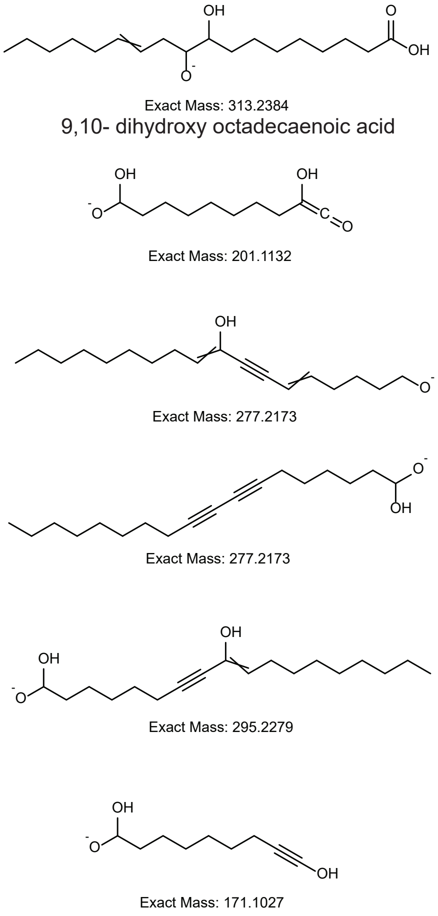

Figure S35. EIC and ms2 fragmentation for 9,10-diHOME. **a)** Extracted ion chromatogram of the 9,10-dihydroxy octadecaenoic acid authentic standard eluting at 5.5 min. **b)** Comparison of the observed fragmentation for m/z= 313.2385 at RT = 5.9 minutes to the oxylipin fragmentation predicted using the CFMID package (Supplemental File 1C). A table quantifying the quality of the ms2 fragmentation match can be found in Supplemental Table 8. **c)** Chemical structure of the proposed parent ion and ms2 fragments that match the authentic standard's modeled fragmentation.

a

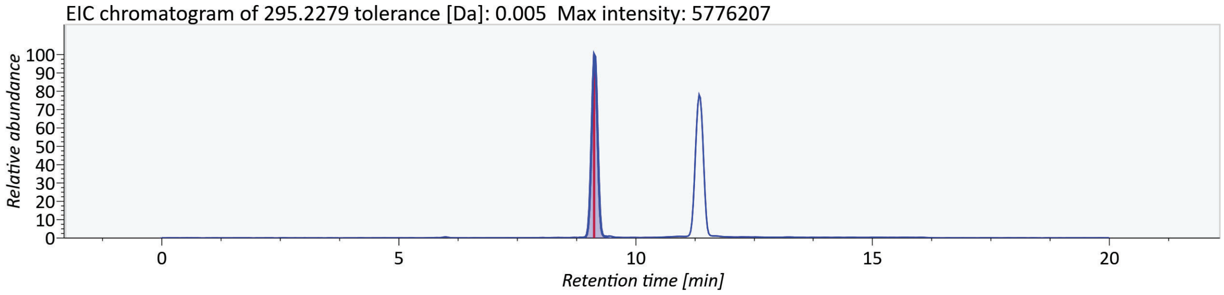

b

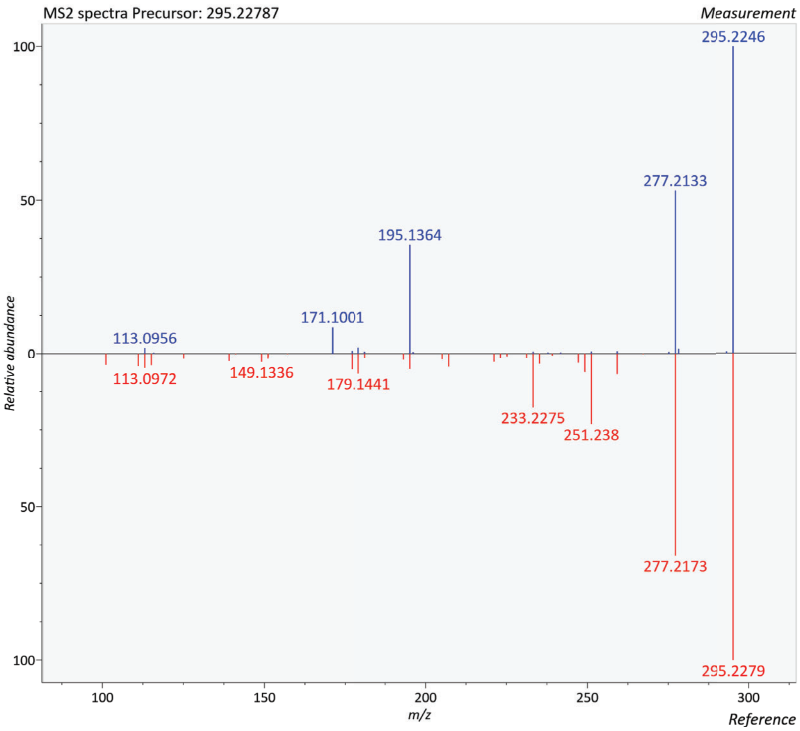

c

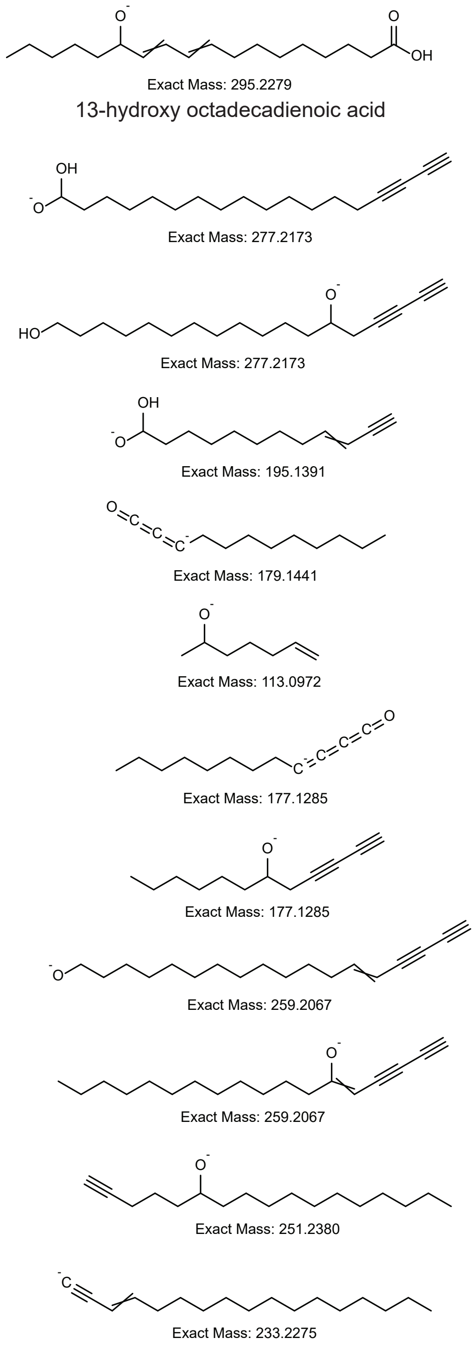

Figure S36. EIC and ms2 fragmentation for 13-HODE. **a)** Extracted ion chromatogram of the 13-hydroxy octadecadienoic acid authentic standard eluting at 9.1 min. **b)** Comparison of the observed fragmentation for m/z= 313.2385 at RT = 9.1 minutes to the oxylipin fragmentation predicted using the CFMID package (Supplemental File 1C). A table quantifying the quality of the ms2 fragmentation match can be found in Supplemental Table 8. **c)** Chemical structure of the proposed parent ion and ms2 fragments that match the authentic standard's modeled fragmentation.

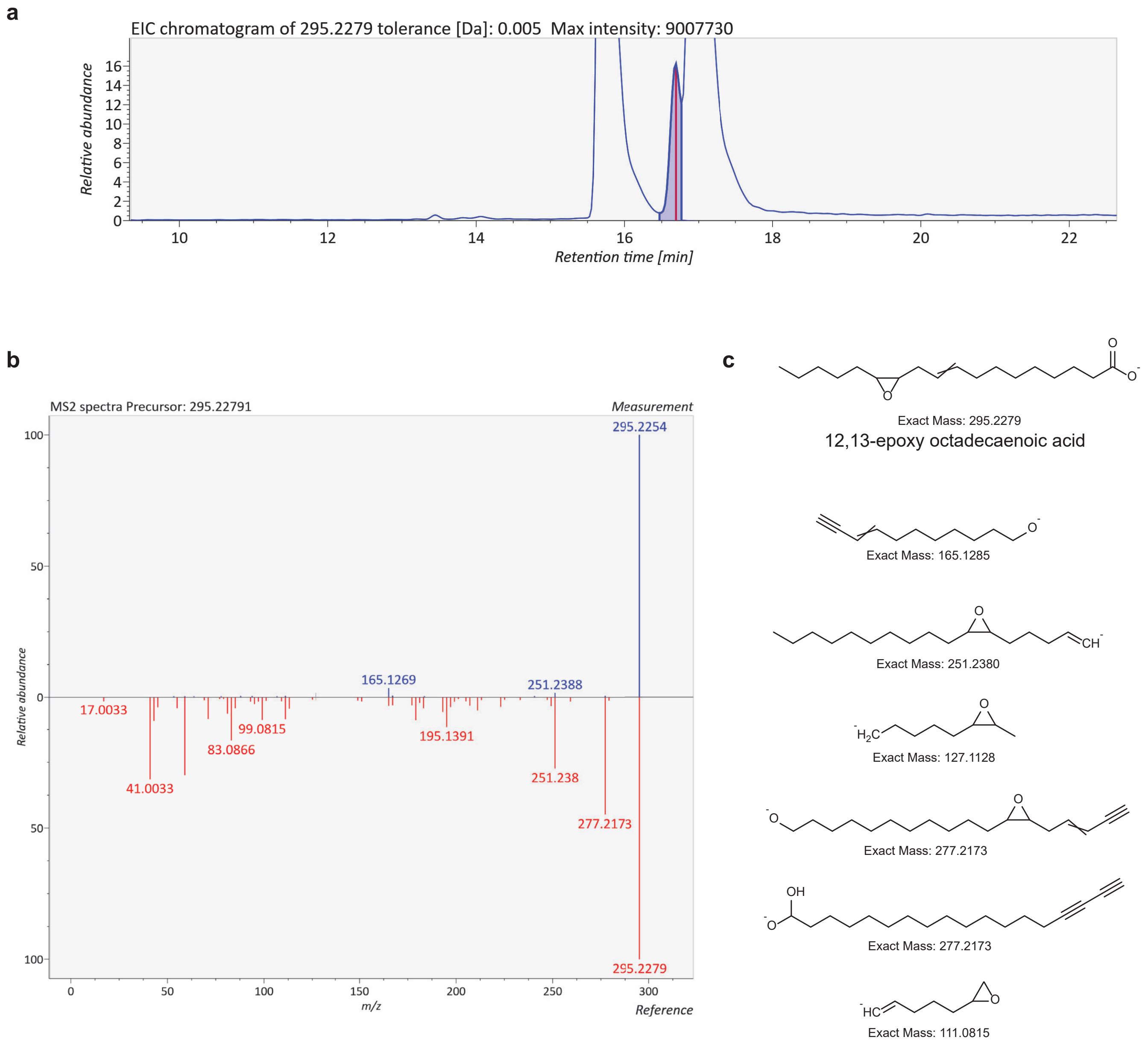

Figure S37. EIC and ms2 fragmentation for 12,13-epOME. **a)** Extracted ion chromatogram of the 12,13-epoxy octadecaenoic acid authentic standard eluting at 16.7 min us the 80/20 method. **b)** Comparison of the observed fragmentation for m/z= 295.2279 at RT = 16.7minutes to the oxylipin fragmentation predicted using the CFMID package
(Supplemental File 1C). A table quantifying the quality of the ms2 fragmentation match can be found in Supplemental Table 8. **c)** Chemical structure of the proposed parent ion and ms2 fragments that match the authentic standard's modeled fragmentation.

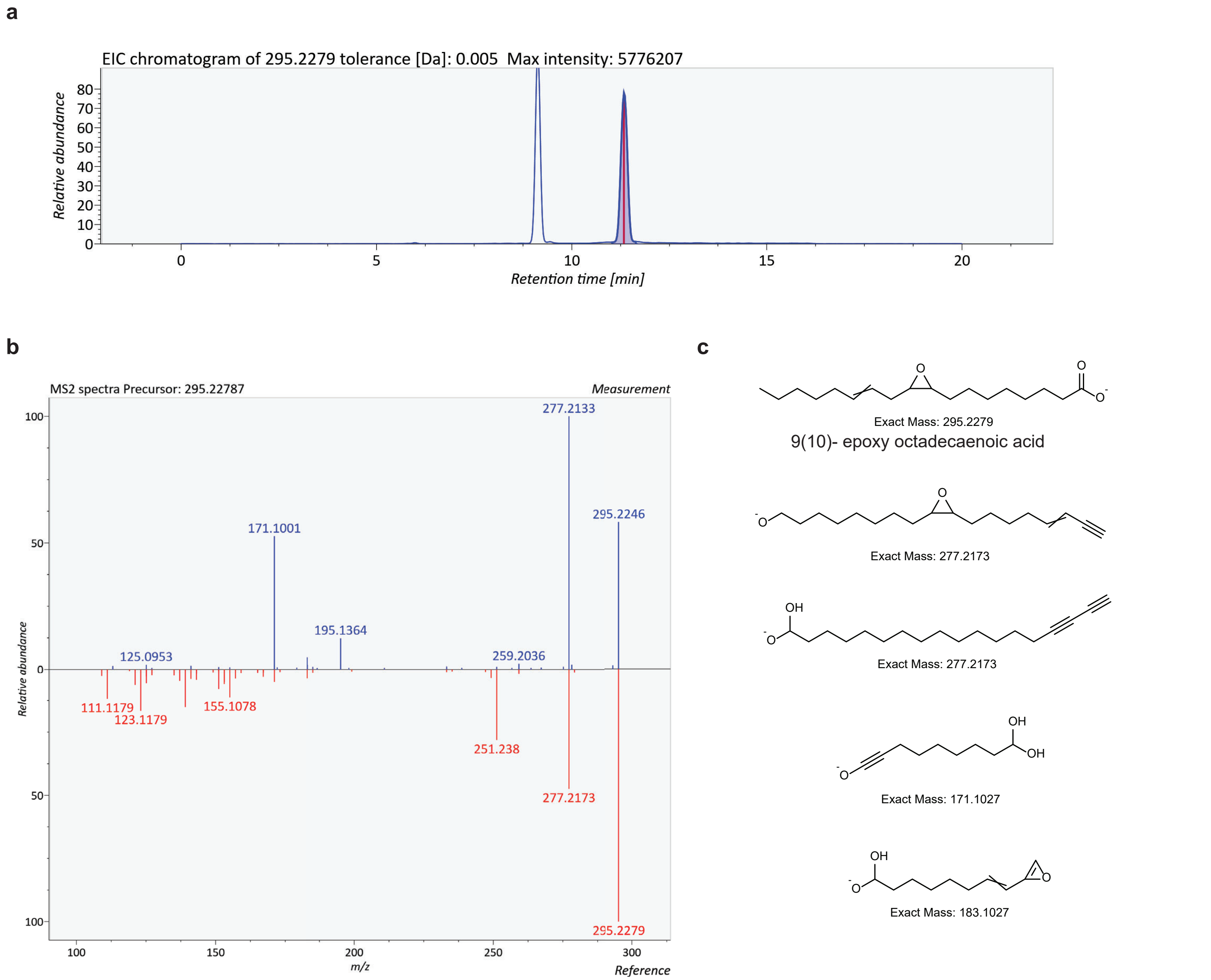

Figure S38. EIC and ms2 fragmentation for 9,10-epOME. **a)** Extracted ion chromatogram of the 9(10)-epoxy octadecaenoic acid authentic standard eluting at 11.13 min. **b)** Comparison of the observed fragmentation for m/z= 295.2279 at RT = 11.13 minutes to the oxylipin fragmentation predicted using the CFMID package (Supplemental File 1C). A table quantifying the quality of the ms2 fragmentation match can be found in Supplemental Table 8. **c)** Chemical structure of the proposed parent ion and ms2 fragments that match the authentic standard's modeled fragmentation.

a

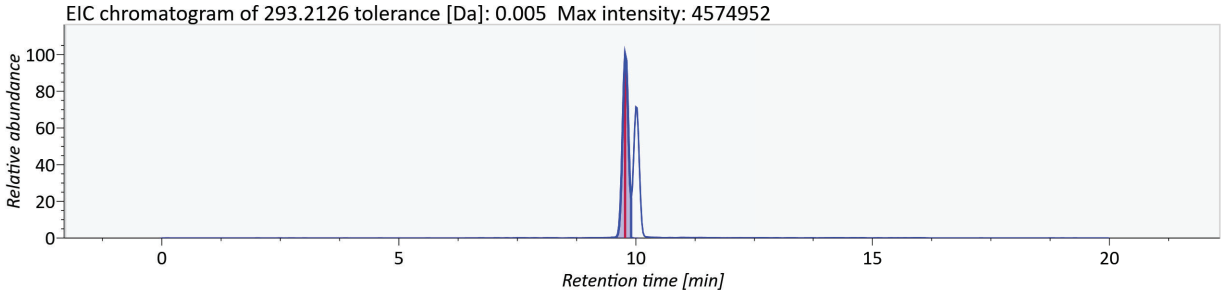

b

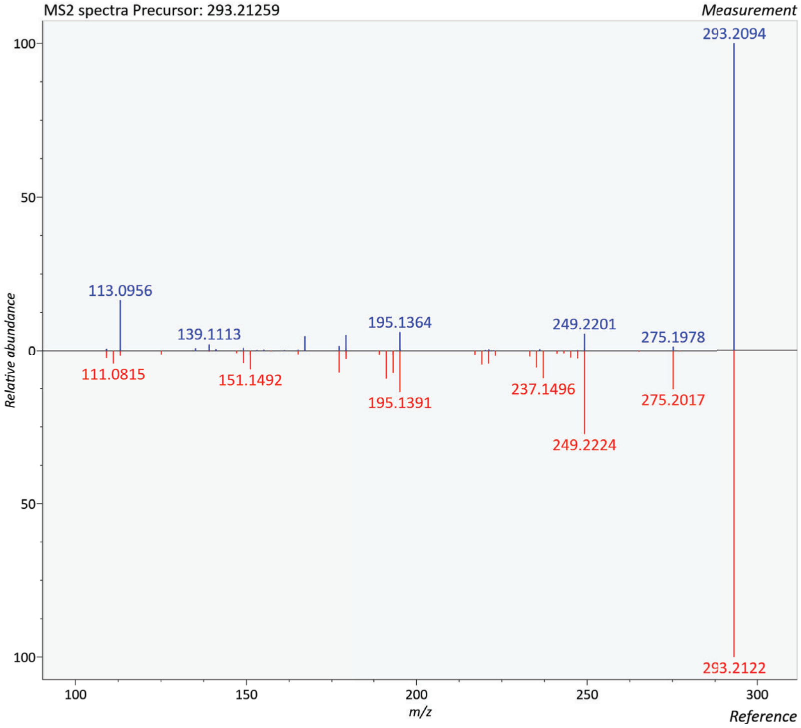

c

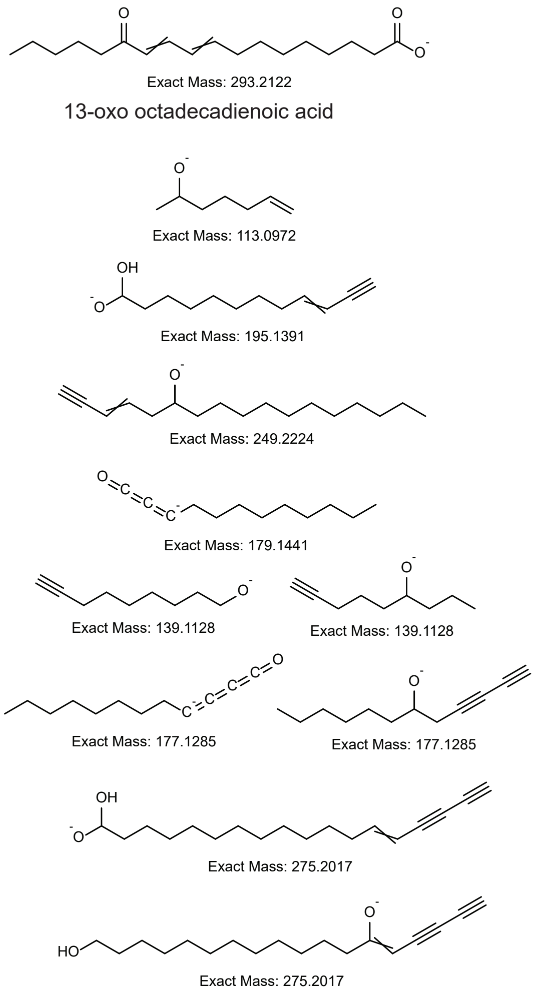

Figure S39. EIC and ms2 fragmentation for 13-oxoODE. **a)** Extracted ion chromatogram of the 13-oxo octadecadienoic acid authentic standard eluting at 9.7 min. **b)** Comparison of the observed fragmentation for m/z= 293.2094 at RT = 9.7 minutes to the oxylipin fragmentation predicted using the CFMID package (Supplemental File 1C). A table quantifying the quality of the ms2 fragmentation match can be found in Supplemental Table 8. **c)** Chemical structure of the proposed parent ion and ms2 fragments that match the authentic standard's modeled fragmentation.

a

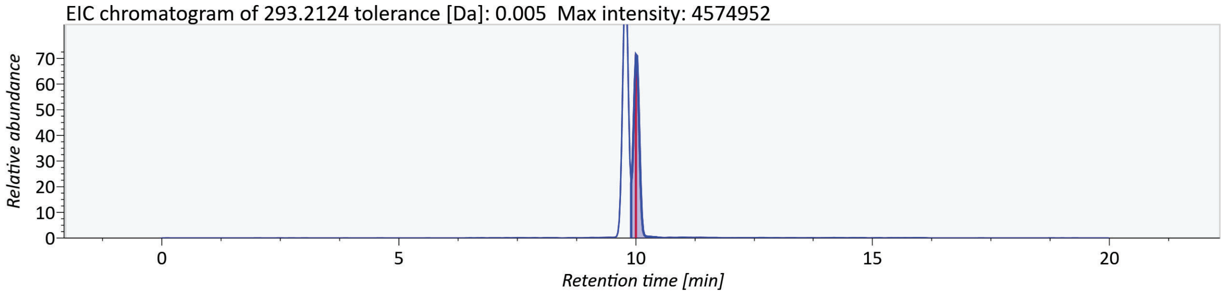

b

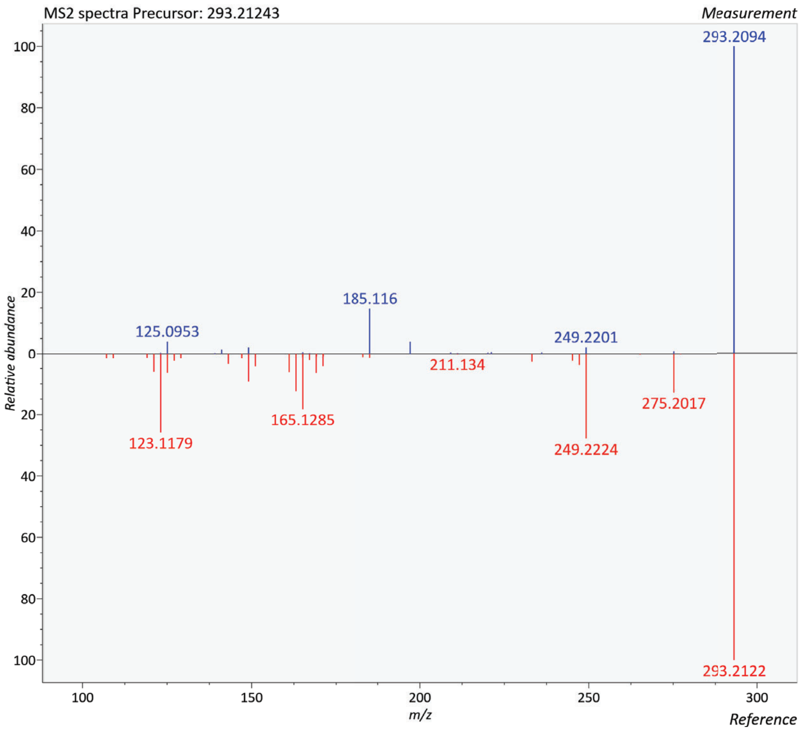

c

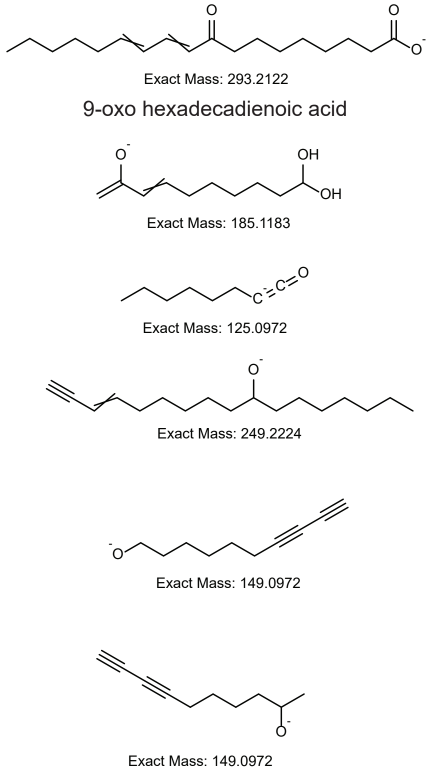

Figure S40. EIC and ms2 fragmentation for 9-oxoODE. **a)** Extracted ion chromatogram of the 9-oxo octadecadienoic acid authentic standard eluting at 10 min. **b)** Comparison of the observed fragmentation for m/z= 293.2094 at RT = 10 minutes to the oxylipin fragmentation predicted using the CFMID package (Supplemental File 1C). A table quantifying the quality of the ms2 fragmentation match can be found in Supplemental Table 8. **c)** Chemical structure of the proposed parent ion and ms2 fragments that match the authentic standard's modeled fragmentation.

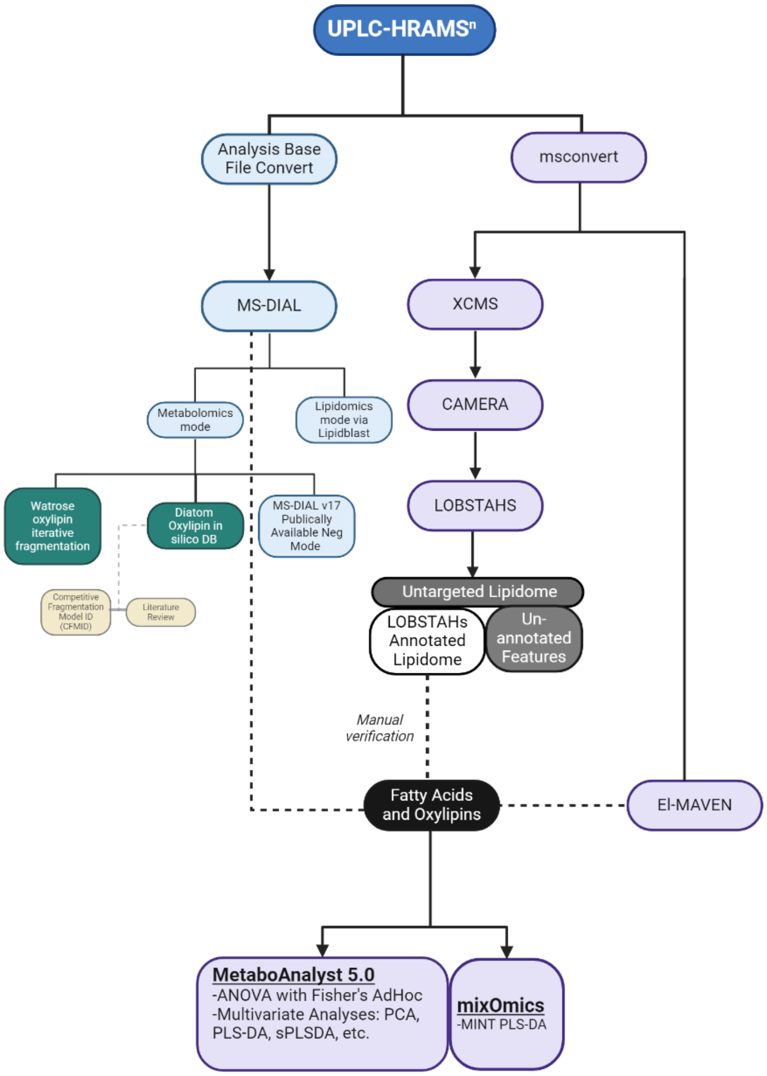

Figure S41. Lipidomic annotation pipeline employed in this study. The purple route was used to analyze the 2016 HPLC-HRAM-MS generated data and the resulting oxylipidome consisting of compounds annotated as free fatty acids and oxylipins were used for statistical analysis laid out in Figure S1. Samples rerun in 2021 using UPLC-HRAM-MS^n were used to improve the annotation of features in the oxylipidome via the Blue-green route in the pipeline.
