# Supplementary material for: Viral Infection Leads to a Unique Suite of Allelopathic Chemical Signals in Three Diatom Host–Virus Pairs
Source: Mar Drugs. 2024 May 17;22(5):228. doi: 10.3390/md22050228 (PMC11123003; doi:10.3390/md22050228)
Supplement: Supplementary file 1 [file marinedrugs-22-00228-s001.zip › Supplemental Table S9 Authentic standard for RT model.pdf]

Table S9. Authentic standards used for the retention time model. Fatty acid, polyunsaturated aldehyde, non-volatile oxylipin standards with the common name or abbreviation, the structural level annotation assigned by the lipidomics pipeline presented here, and the retention time (RT) of each compound. Comparisons to the RT of the authentic standards were used to assign putative annotations to features in the dissolved lipidome.

Authentic Standard	Common Abbreviation/ Name	Structural Level Annotation	RT
Octanoic acid	--	FFA 8:0	2.65
Arachidonic acid	AA	FFA 20:4	8.09
Docosahexaenoic acid	DHA	FFA 22:6	7.71
9-Hydroperoxy octadecadienoic acid	9-HpODE	NVO 18:2 2O	5.44
13-Hydroperoxy octadecadienoic acid	13-HpODE	NVO 18:2 2O	5.44
9-Hydroxy octadecatrienoic acid	9-HOTrE	NVO 18:3 1O	4.02
13-Hydroperoxy octadecatrienoic acid	13-HpOTrE	NVO 18:3 2O	4.65
9-Hydroperoxy octadecatrienoic acid	9-HpOTrE	NVO 18:3 2O	4.28
15-Hydroxy eicosatrienoic acid	15-HETrE	NVO 20:3 1O	6.11
15-Hydroxy eicosatetraenoic acid	15-HETE	NVO 20:4 1O	5.43
5-Hydroperoxy eicosatetraenoic acid	5-HpETE	NVO 20:4 2O	5.01
12-Hydroperoxy eicosatetraenoic acid	12-HpETE	NVO 20:4 2O	5.95
15-Hydroperoxy eicosatetraenoic acid	15-HpETE	NVO 20:4 2O	5.73
5,6-Dihydroxy eicosatetraenoic acid	5,6-diHETE	NVO 20:4 2O	3.48
14,15- Dihydroxy eicosatetraenoic acid	14,15-diHETE	NVO 20:4 2O	3.00
5,12- Dihydroxy eicosatetraenoic acid	Leukotriene B4	NVO 20:4 2O	2.89
15-Hydroxy eicosapentaenoic acid	15-HEPE	NVO 20:5 1O	4.56
5,6,15- Trihydroxy eicosapentaenoic acid	Lipoxin A5	NVO 20:5 3O	1.72
Heptadienal	--	PUA 7:2	1.90
Octadienal	--	PUA 8:2	2.28
Nonadienal	--	PUA 9:2	2.69
Decadienal	DD	PUA 10:2	3.65
